# Major Achievements in the Design of Quadruplex-Interactive Small Molecules

**DOI:** 10.3390/ph15030300

**Published:** 2022-02-28

**Authors:** Eduarda Mendes, Israa M. Aljnadi, Bárbara Bahls, Bruno L. Victor, Alexandra Paulo

**Affiliations:** 1 Faculty of Pharmacy, Research Institute for Medicines (iMed.Ulisboa), Universidade de Lisboa, 1649-003 Lisbon, Portugal; ermendes@ff.ulisboa.pt (E.M.); israa.aljnadi@campus.ul.pt (I.M.A.); barbara.bruni@edu.ulisboa.pt (B.B.); 2 Faculty of Sciences, BioISI, Biosystems and Integrative Sciences Institute, Universidade de Lisboa, 1749-016 Lisbon, Portugal; blvictor@fc.ul.pt

**Keywords:** nucleic acids, G-quadruplex, i-Motif, organic molecules, drug design, cancer, virus, bacteria, malaria

## Abstract

Organic small molecules that can recognize and bind to G-quadruplex and i-Motif nucleic acids have great potential as selective drugs or as tools in drug target discovery programs, or even in the development of nanodevices for medical diagnosis. Hundreds of quadruplex-interactive small molecules have been reported, and the challenges in their design vary with the intended application. Herein, we survey the major achievements on the therapeutic potential of such quadruplex ligands, their mode of binding, effects upon interaction with quadruplexes, and consider the opportunities and challenges for their exploitation in drug discovery.

## 1. Introduction

The association of nucleic acid sequences into a wide range of secondary and higher-order structures is nowadays well known to be of crucial importance in genome regulation in association with proteins. The first evidence of the formation of a helical quadruplex structure due to the self-assembly of polyguanilic acid came in 1962 [1] and later in the 1980s Sen and Gilbert [2] reported the formation of a four-stranded structure from a guanine-rich DNA sequence, the G-quadruplex (G4). But it was only in the early 1990s that the G4 three-dimensional structure was solved by NMR spectroscopy [3,4] and X-ray crystallography [5]. Since then, G4s were shown to form not only in vitro but also in cells [6,7], in the entire genome, even though quadruplex-forming sequences are particularly enriched in telomeres, gene promoters, and other untranslated regions of the genome [8]. In the past two decades, G4s have generated a huge interest among the scientific community due to their versatile applicability in nanotechnology and medicinal fields. Another important DNA quadruplex (or tetraplex) is formed by the self-assembly of cytosine-rich sequences into an intercalated-double helix motif, known as i-Motif (iM).

This structural arrangement was first reported in the 1990s based on NMR [9] and X-ray crystallographic [10] studies in acidic conditions. Due to these requirements, iMs were for long considered to be unable to form stable structures in cells. Nevertheless, they have been exploited in nanotechnology, particularly as pH-nanosensors [11]. However, more recently, iMs were detected in nuclei of living mammalian cells by NMR [12], where they play an important role, together with the complementary G4-forming sequences, in the regulation of gene transcription [13].

In this overview, we summarize the major discoveries reported up to date on the potential of quadruplex-interactive organic small molecules as therapeutic agents, their mode of binding based on G4-ligand complexes determined by NMR or crystallography, as well as the main ligand-induced effects on quadruplex structures (G4s or iMs) and how these can be exploited. Based on the reported achievements, we will discuss the opportunities and challenges ahead. Metal-based quadruplex-interactive compounds have also been developed and shown relevant properties that can be exploited in the development of probes, diagnostic tools, or therapeutic agents, as recently reviewed elsewhere [14,15].

### 1.1. Structures of G-Quadruplexes and i-Motifs

**Figure 1:**
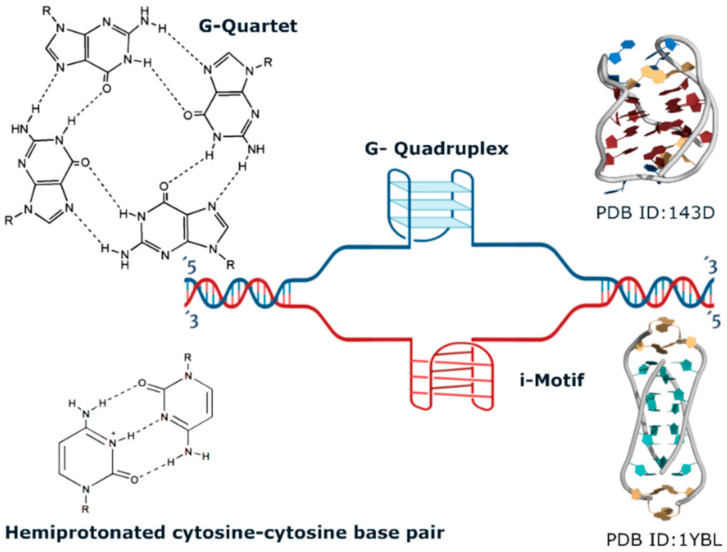
Schematic representation of DNA G-quadruplexes and i-Motifs. Top: G-quadruplexes (G4s) are formed within guanine-enriched regions of the genome. Four guanines bind through hydrogen bonding and arrange in-plane to form a G-quartet that stacks on top of each other to form a G4 (e.g., PDB ID 143D). Bottom: i-Motifs are formed in a cytosine enriched region via hydrogen bonding of hemiprotonated cytosine–cytosine base pairs (e.g., PDB ID 1YBL).

G4s and iMs are four-stranded structures formed by nucleic acid sequences containing stretches of consecutive guanines or cytosines, respectively (Figure 1).

The stacking of two or more square units formed by four in-plane guanines linked through Hoogsteen hydrogen bonds (the G-quartet or G-tetrad) originates the helical structure of the G4 [16]. More recently, quadruplexes formed by mixed quartets, for example, composed of guanines and cytosines, have also been reported [17,18]. The stability of G-quadruplexes arises from hydrogen bonding between intraquartet guanines, interquartet π–π stacking interactions, and by additional coordination of guanines O6 with cations. The following trend for G4 stabilization by cations has been proposed: K^+^ > NH4^+^, Na^+^ > Mg^2+^ > Li^+^, with lithium ions having almost no effect on G4 stabilization [19].

G4s can be formed from intramolecular folding of a DNA or RNA single strand with the general sequence GaXbGaXcGaXdGa, where ‘a’ represents the number of consecutive guanines (usually ≥3) in each G-stretch and Xb, Xc, and Xd represent any combination of nucleobases (including guanines) forming the loops holding together the G-quartets. G4s can also form from the intermolecular association between guanines of two or four different strands. Intramolecular RNA-G4s are usually more stable than correspondent DNA-G4s [20].

**Figure 2:**
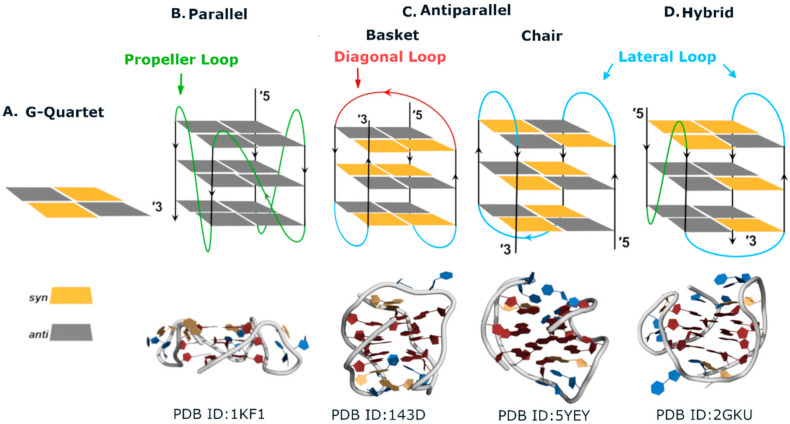
Topologies of intramolecular G-quadruplexes (G4). (**A**) Illustration of a G-quartet where squares represent guanine residues. Colour scheme of guanosine glycosidic bond angles: *anti* is represented in grey and *syn* in yellow. (**B**) Top: illustration of a parallel G4 formed by three stacked G-quartets linked by propeller loops. Bottom: representation of the structure of the telomeric parallel G4 determined in solution (PDB ID 1kf1). (**C**) Top: illustration of G4s arranged in antiparallel topologies. In the “chair-type” conformation, all loops are lateral, whereas lateral and diagonal loops tie up the “basket-type” conformation. Bottom: representation of an antiparallel (basket) structure (PDB ID 143D) and an antiparallel (chair) structure (PDB ID 5YEY). (**D**) Top: illustration of a hybrid G4 in which the propeller and lateral loops link G-quartets. Bottom: representation of the structure of a hybrid G4 (PDB: 2GKU).

G4s are polymorphic and dynamic structures, folding into different topologies depending not only on the nucleotide sequence but also on environmental conditions, such as the ionic strength of the medium, the identity of the metal cation, or the presence of crowding agents [21,22,23,24]. G4 topologies may be classified according to strand polarities (orientations) into parallel, antiparallel, or hybrid (Figure 2). 

Strand polarity also determines loop arrangements in an intramolecular G4. Adjacent parallel strands are linked by propeller (or strand-reversal) loops connecting the bottom G-quartet with the top G-quartet, whereas antiparallel strands can be linked by diagonal or lateral loops [16]. In addition, loop size can influence the folding topology of an intramolecular G4-forming sequence. Single nucleotide loops seem to be too short to form a diagonal loop, thus disfavoring an antiparallel topology [25]. Guanine glycosidic angles and groove widths are also determined by strands polarities. Parallel quadruplexes have all glycosidic angles in an *anti* conformation, while antiparallel and hybrid G4s have both *syn* and *anti* guanosines (Figure 2B–D) [16,26].

Most therapeutically relevant DNA G4s, like those occurring in human telomeres and promoter regions of oncogenes, are intramolecular G4s. Differently from telomeric G4 DNA that has a repeat sequence of six nucleotides (TTAGGG) [27,28], the G4-forming sequences present in gene promoters are diverse and often contain more than four stretches of guanines. Thus, a given sequence can form multiple G4s by varying the combination of G-stretches. For example, *c-MYC* promoter G4 sequence Pu27 has five G-stretches, and it can fold into multiple species [29,30,31], being the G4 involving G-stretches 2-3-4-5 the more stable and prevalent species [30]. It has been suggested that the presence of more than the required four stretches of consecutive guanines represents an evolution of nature to deal with genomic instability due to oxidative stress [32,33]. When participating in G4 quartets, oxidized guanines are not substrates of base excision repair enzymes. Thus, a fifth G-stretch is probably necessary to replace the damaged G-stretch by promoting the extrusion of that G-stretch into a loop where oxidized guanines can be repaired. In addition, more than one sequence able to form a stable G4 can be found upstream of the transcription start sites of these genes, for example, in the case of *c-KIT* [34].

Intramolecular G4s found in promoter genes fold, in general, into parallel topologies, but even parallel G4s can have 3D structures very different from each other, as discussed elsewhere [35]. Interestingly, very recently, it has been found that a G-rich sequence in MDM2 oncogene promoter can fold into a stable four-quartet antiparallel G4 structure [36]. Structural diversity arises not only from different combinations of G-stretches (as in *c-Myc* promoter), loops sequences, and length, but also from involving loops’ guanine residues into G-quartets as in the case of ckit1 (PDB ID 4WO2) [37].

i-Motifs (iMs) are formed by the intercalation of hemiprotonated cytosine–cytosine base pairs (C-C+), linked through Hoogsteen H-bonds (Figure 1). The i-Motifs can also be formed from one, two, or four DNA strands but have a very different geometry from G4. Although more extended than a single DNA duplex, they can be rationalized as two intercalated duplexes and present two extensive grooves and two very narrow grooves. Polymorphism in iM, contrary to G4, is very limited due to low variability in strands orientation since the two diametrically distant strands must be parallel to each other [9]. Also, conditions facilitating iM formation are different from those stabilizing G4. iMs are very sensitive to pH, whereas cations do not affect iM formation and stability. Since cytosine–cytosine pairing requires the protonation of N3 of one base, the pH leading to maximum iM stability is around cytosine pKa (4–5) [38]. On the other hand, the formation and stabilization of both G4 and iM are favored by crowding conditions [39,40,41].

### 1.2. Quadruplexes as Therapeutic Targets

G4-forming nucleic acid sequences were identified in telomeres and other regions of the genome of many organisms, including humans [42,43,44], bacteria [45,46], viruses [47,48,49], plants [50] and parasites [51,52,53]. Most often, the genomes were first screened using informatic tools, such as the G4 Hunter algorithm [54,55], followed by biophysical, biochemical and bio-imaging techniques to confirm the G4 structures and their presence in cells [43]. In a fantastic recent work, the group of Balasubramanian used a G4-sequencing method to map the genome of 12 species, including bacteria, yeast, human pathogenic parasites, and mammals (human and mouse) [56]. This study confirmed many of the conclusions of the previous bioinformatic studies. Still, it also reached the interesting conclusion that the distribution of non-telomeric G4s in the genome seems not to follow an evolutionary chain logic. Experimental evidence for the formation of G4s in human cells has already been obtained using in vivo NMR [57], G4-specific antibodies, and chemical probes [7,58,59,60]. In a single-molecule fluorescence imaging study, Di Antonio and Balasumabranian showed the dynamic nature of G4 formation in living cells and demonstrated that it is cell-cycle-dependent and can be disrupted by chemicals inhibiting transcription and replication [61], probing the potential of G4s as drug targets. In the past years, several studies have shown the potential of quadruplex-interactive small molecules for the treatment of, particularly, cancer and viruses, but also for bacterial and parasitic infections. More recently, DNA and RNA G4s have also been proposed as drug targets for neurological diseases [62,63,64]. 

#### 1.2.1. Quadruplexes in Cancer

In the case of the human genome, computer analysis revealed a prevalence of this type of sequences in telomeres and promoter regions of proto-oncogenes, and the involvement of G4s in the regulation of epigenetic, replication, transcription, and translation processes has been demonstrated by several studies, recently reviewed elsewhere [65,66,67]. It has also been shown that G4s are structures that occur more frequently in areas of the genome with high transcription rates and in cells with increased proliferation rates, such as cancer cells [68]. All of this evidence, coupled with the fact that in many cases (but not all), G4s negatively control the transcription of well-known oncogenes, such as *c-MYC* [69] and *k-RAS* [70], makes G4s an attractive and promising target in oncotherapy [71,72]. However, it must be noted that many promoter regions can form more than one G4 structure that work together, in a dynamic equilibrium, to regulate gene expression. For example, the *c-KIT* promoter has three adjacent G-rich domains able to form three G4 structures, named kit1, kit2 and kit*. The G4-kit* is a known SP1 binding site that works in close relation with G4-kit2 [73,74] to induce the expression of the proto-oncogene, whereas G4-kit1 formation leads to decreased gene expression [34].

Many G4-interactive organic small molecules, with most of them able to stabilize G4s in vitro, have also been shown to possess antiproliferative activity in cancer cell cultures, with concomitant induction of DNA damage and apoptosis, hallmarks of G4 ligands inducing cellular effects [75]. Some of these molecules have also shown therapeutic activity in xenograph models of cancer, but only three have so far progressed to clinical trials (see Table 1). 

**Figure 3:**
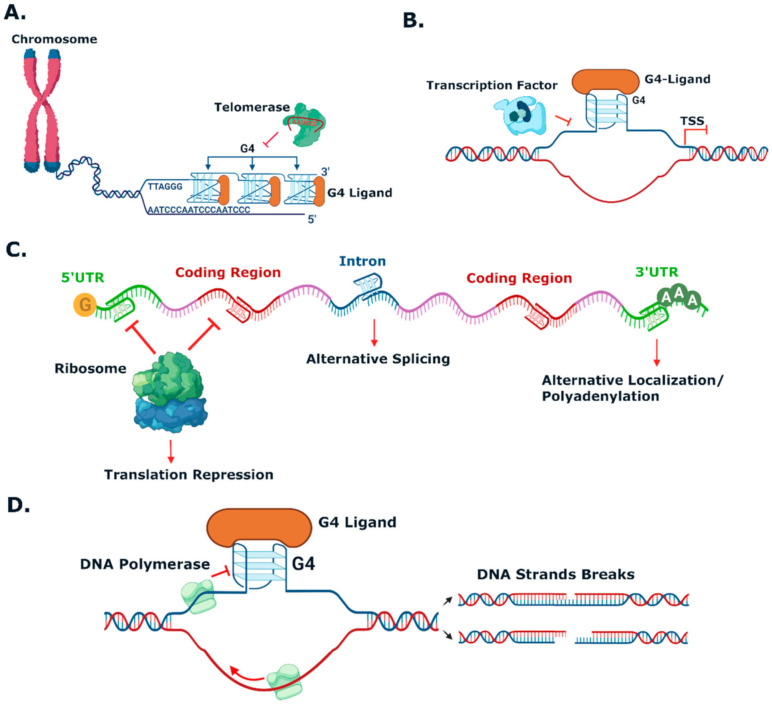
Cellular processes that G4 ligands can target. (**A**) Telomere maintenance. Enriched-guanine telomeres may form G4s at 3′ overhang. Telomeric G4 blocks the telomerase and alters the telomere’s elongation and maintenance. (**B**) Oncogene promoter regulation. Formation of G4s within the promoter region usually occurs close to the transcription start site (TSS) and may block transcription factors binding, resulting in downregulation of oncogene expression. (**C**) Translation and mRNA splicing. G4 formation can occur on mRNA strands at different sites causing variations in gene translation. G4s at 5′ UTR and coding regions block ribosomal activity leading to translation inhibition; G4s within intron leads to alternative splicing, and G4s at 3′ UTR causes translation inhibition and alternative mRNA localization. (**D**) DNA replication. In cell division, the process of DNA replication can be paused due to G4 formation, leading to genomic instability. DNA strand containing G4s will be scanned with gaps by DNA polymerase. These gaps in the new strand lead to DNA double-strand breaks over the following cell division event.

Figure 3 schematizes the main cellular mechanisms by which it is believed that G4-stabilizing small molecules induce cancer cells death [76,77,78] and Table 1 summarizes the effects in cancer cell lines and in-vivo models of selected G4-interactive small molecules. First studies on the exploitation of G4s as therapeutic targets for cancer focused on the potential of inhibiting telomerase activity by inducing and stabilizing the G4s in the telomere 3′ end (Figure 3A). 

**Figure 4:**
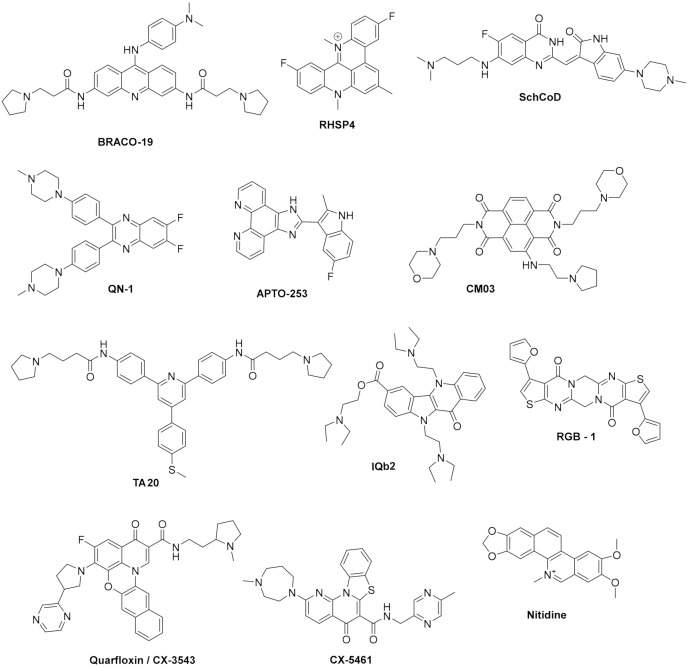
Structures of G4 ligands with anticancer activity.

Telomerase is a reverse transcriptase that is overexpressed in cancer cells. It plays a central role in cells immortalization by catalysing the synthesis of telomere DNA repeats at 3′end, thus maintaining the telomere length and avoiding cell senescence. However, it was found that small molecules targeting G4s in telomeres, such as the acridine derivatives depicted in Figure 4 **BRACO-19** [79] and **RHSP4** [80], and the quinazolinone **SchCoD** (a derivative of the natural product schizocommunin; Figure 4) [81] also induced a DNA damage response due to telomere uncapping. 

Another approach being exploited is the inhibition of oncogene expression by targeting DNA G4s in promoters (Figure 3B) or mRNA G4s in 5′ UTRs (Figure 3C). DNA G4 motifs have been found in promoter regions, usually within 1kb upstream of the transcription starting site (TSS), of many cancer-related genes, such as *c-MYC*, *c-KIT*, *HIF*, *BCL-2*, *RET*, *k-RAS*, *h-RAS*, *HSP90*, *b-RAF*, *VEGF*, *hTERT* [35,72] and *MDM2* [36]. Several studies have shown that G-rich sequences are recognition sites for transcription factors, such as Sp1 and MAZ, [82,83] among others [66], which implies that they play an important role in transcription regulation. In the past decade, it has been generally accepted that G4 formation in many oncogene promoters can inhibit the transcription process, possibly by blocking the binding of transcription factors to the promoter and interfering with transcription initiation by RNA polymerase [76,84]. Resolution of these secondary DNA structures by helicases is then necessary to restore the transcription activity [85].

Many small molecules able to stabilize in vitro the DNA G4 structures found in the promoter regions of oncogenes were also shown to have anticancer activity in cell lines and in xenograft mice models of cancer, as well as having the capacity to downregulate the transcription of certain G4-containing genes involved in cancer cells proliferation [35,86,87]. Most of the G4 ligands, like the selected ones presented in Table 1, were reported as having a preference of binding and in-vitro stabilization of a specific G4 structure. That is the case of the quinoxaline derivative **QN-1** (Figure 4) [88] and the phenanthroline **APTO-253** (Figure 4) [89,90] for the G4-forming sequences in the *c-MYC* promoter. In cancer cell lines overexpressing c-MYC, these compounds downregulated *c-MYC* expression, upregulated tumor suppressor genes (KLF4 and p53), induced DNA damage, apoptosis, and cell-cycle arrest in the G0–G1 phase. However, it is unlikely that these compounds only target the *c-MYC* promoter G4. The cytotoxic events observed upon cell treatment with **APTO-253** can also be explained by the DNA- damage elicited by the stabilization of DNA G4 during transcription [91] and replication (Figure 3D) [65]. In fact, whole-genome sequencing analysis of pancreatic cancer cells treated with the non-selective G4 stabilizers, the naphthalene diimide **CM03** (Figure 4) [92], and the transcriptome studies with the triaryl-pyridine **TA20** (Figure 4) [93], support a poly-G4-targeting mechanism of action for these compounds. Interestingly, in both studies, genes containing G4-forming sequences in their promoters are preferentially downregulated, supporting the rationale of targeting G4s in oncogene promoters with G4-stabilizers. 

**Figure 5:**
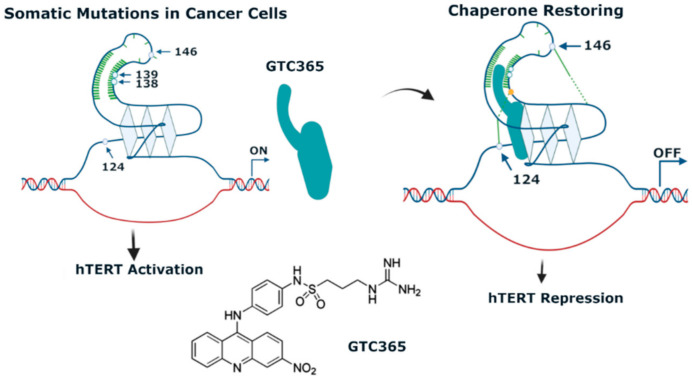
Mechanism of *hTERT* regulation by **GTC365**. Human telomerase reverse transcriptase (hTERT) is transcriptionally silenced in normal cells. Mutations in *hTERT* promoter have been found to occur in many cancer types, which activate *hTERT* transcription. The acridine GTC365 partially restores the wild-type G4-folding leading to *hTERT* transcription repression.

Moreover, not all genes containing G4 structures in promoters are equally downregulated by the same G4-stabilizer in each cell line. An example of this has been provided by a study with the indoloquinoline **IQb2** (Figure 4) [94]. This molecule was shown to be equally potent, stabilizing in-vitro the 21nt G4 structures present in *k-RAS* and *HSP90* promoters. However, in HCT116 colon cancer cells, which express a mutated *k-RAS*, **IQb2** significantly downregulated transcription of this oncogene but had no significant impact on the transcription of the *HSP90* gene. Moreover, in a non-cancer cell line expressing wild-type *k-RAS* and *HSP90* genes, **IQb2** at equitoxic concentrations (IC_50_ concentrations) had minimal effect on the expression of both genes [94]. Another interesting approach to selectively target a given promoter G4 is that developed by Hurley’s lab. The acridine **GTC-365** was designed to target the G-quartet and the duplex stem-loop close to the G4 structure of the *hTERT* promoter (Figure 5) [95]. Stabilization of this stem-loop helps restore the wild-type tertiary structure from the promoter DNA mutated sequence, resulting in downregulation of *hTERT* transcription and induction of cell death through many mechanisms leading to apoptosis and cell senescence due to reduction of telomerase activity.

G4s can also be formed in different sites of mRNA strand, affecting not only translation but also mRNA splicing and translocation (Figure 3C). G4s at 5′ UTR region can interfere with recruitment or scanning of 43S pre-initiation complex and consequently inhibit translation, whereas G4s at open reading frames can also repress translation by stalling ribosomes; G4s within introns can lead to alternative splicing; and G4s at 3′ UTR can interfere with mRNA maturation, translocation and translation [20,96]. Examples of small molecules targeting RNA G4s are fewer. One such example is that of **RGB-1** (Figure 4), an RNA G4 selective ligand found through a reverse transcriptase-based screening method using the telomeric repeat-containing RNA (TERRA) G4-forming sequence. This molecule was shown to decrease translation of mRNA containing G4 structures, both in-vitro and in cancer cells [77].

**Table 1:** Effects of selected G4 ligands in in-vitro and in-vivo cancer models. Structures are depicted in Figure 4.

G4 Ligand (Chemotype)	Target in Cancer Cells	Effects	Refs.
**BRACO-19** (acridine)	Telomere G4 in glioblastoma cells	Induces telomere uncapping; inhibits telomerase activity; induces DNA damage, apoptosis and senescence.	[79]
**RHSP4** (acridinium)	Telomere G4 in glioblastoma cells	Radiosensitizing agent in a glioblastoma multiforme xenograft model, inducing telomere dysfunction.	[80]
**SchCoD** (quinazolinone)	Telomere G4 in cervical squamous cancer	It induces telomere uncapping, leading to a DNA damage response and inhibits tumor growth in a xenograft model.	[81]
**QN-1** (quinoxaline)	*c-MYC* promoter G4 in triple-negative breast cancer	Downregulation of c-MYC transcription in vitro and in vivo. Anticancer activity in a xenograft model.	[88]
**APTO-253** (phenanthroline)	*c-MYC* promoter G4 in acute myeloid leukemia	Inhibits c-Myc expression, induces cell cycle arrest and apoptosis in acute myeloid leukemia cells; induces the Krüppel-like factor 4 (KLF4) tumor suppressor.	[89,90]
		In Phase I clinical trials.	[97]
**IQb2** (indoloquinoline)	*k-RAS* promoter G4 in colon cancer cells	Decrease wild-type k-RAS promoter activity; preferentially downregulation of k-RAS expression compared to other G4-containing genes; induces apoptosis and expression of tumor suppressor p53 in HCT116 cell line.	[94]
**GTC365** (acridine)	*hTERT* promoter G4 in breast cancer cells	Binds to G-quartet and duplex stem loop of G4, restoring wild-type folding topology; Induces apoptosis and senescence in cancer cells.	[95]
**TA20** (triarylpyridine)	Several DNA G4 in cervical cancer cells	Stabilizes several G4s in vitro. Transcriptome analysis of treated cancer cells revealed that most affected genes are those related with DNA damage, cell growth and autophagy; genes enriched in G4-forming sequences are preferentially downregulated.	[93]
**CM03** (naphthalene diimide)	Several DNA G4 in pancreatic cancer	Stabilizes several G4s in vitro and increases G4 foci in treated cells; Transcriptome analysis indicates a preferential downregulation of genes with G4-forming sequences in promoters and implicated in cancer cells survival, development of metastasis and drug resistance; Anticancer activity in a xenograft model.	[92]
**RGB-1** (thienopyrimidinone)	5′-UTR G4 of NRAS mRNA in breast cancer	Downregulation of *NRAS* expression in cancer cell lines.	[77]
**Quarfloxin/CX-3543** (quinolone)	Ribosomal DNA G4 in Carcinoid/neuroendocrine tumors	Inhibits rRNA biogenesis; induces DNA damage and apoptosis.	[98]
		Reached Phase II clinical trials.	[99]
**CX-5461** (quinolone)	Ribosomal DNA G4 in BRCA1/2-deficient tumors	Inhibits RNA polymerase I and Topoisomerase II.	[100,101]
		Induces G4-mediated DNA damage.	[102]
		In Phase I Clinical trials.	[103]

Ribosomal DNA G4s can also be targeted with small molecules. The quinolone derivative **CX3543**, also known as Quarfloxin (Figure 4), was the first G4-ligand to reach clinical trials to treat cancer [99]. This molecule was rationally developed from the antibiotic norfloxin to have increased selectivity to G4 structures over duplex DNA and to be devoid of gyrase or topoisomerase II poisoning activity. Initially thought to target *c-MYC* promoter G4s, it was later demonstrated that **CX3543** accumulates in the nucleoli, where it binds to ribosomal DNA G4, inhibiting the activity of RNA-polymerase I and blocking the binding of nucleolin to these G4s. The cellular response is a redistribution of nucleolin into the nucleoplasm, which leads to several events, including the binding of nucleolin to *c-MYC* promoter G4 and inhibition of *c-MYC* transcription [98]. This indirect mechanism can explain the observed *c-MYC* transcription inhibition in HCT116 colorectal tumor tissues taken from mice treated with **CX3543** [104]. More recently, the related quinolone **CX-5461** (Figure 4) entered phase I clinical trials for BRCA1 and BRCA2-deficient breast cancer [103]. The anticancer mechanism of action of **CX-5461** is thought to be also by inhibition of RNA-polymerase I [100] and induction of G4-mediated DNA damage [102]. However, a very recent study indicates that, in fact, its primary mechanism of action is by topoisomerase II poisoning [101]. In a first approach and looking to the structures of both compounds, it seems that the rationale used in the development of **CX-5461**, that is, of reduction of molecule aromatic core and side chains sizes, eventually to comply with conventional “drug-like” properties, led to the loss of the selectivity to G4 achieved with parent compound **CX-3543**.

**Figure 6:**
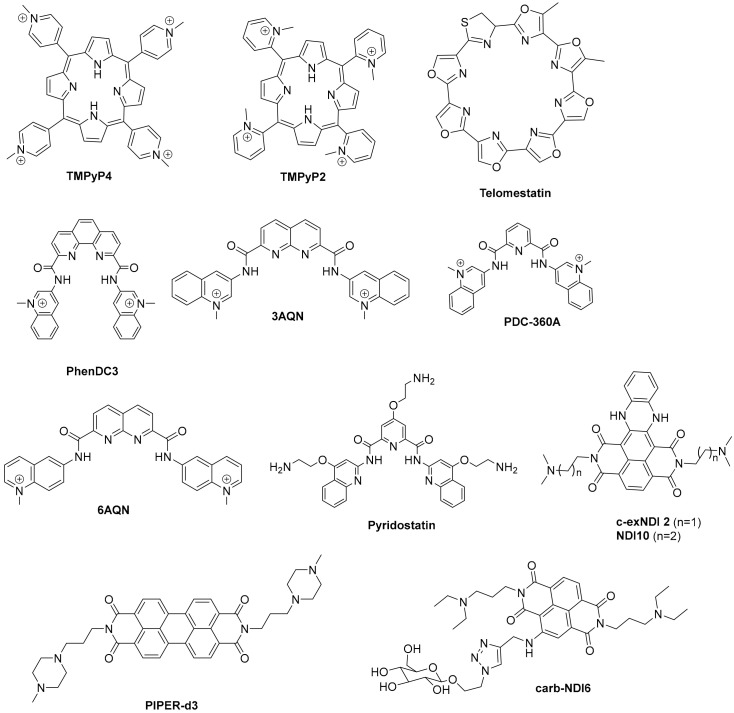
Structures of G4 ligands mentioned in Table 2 and Table 3.

Moreover, G4 stabilizers such as **pyridostatin** and **PhenDC3** (Figure 6) have also been shown to promote type I interferon production and innate immune gene activation, which could be helpful in combination immunotherapies for unresponsive tumors [105].

The complementary C-rich sequences in the promoters can also form quadruplex structures, the iMs, that, in coordination with G4s, regulate gene transcription. The presence of iMs in nuclei of human cells was demonstrated by NMR studies [12] and using fluorescent antibodies [106]. The evidence supporting the activator or repressor role of iMs on gene transcription has been very recently reviewed by Kendrick [13]. The data suggest that they act differently depending on the specific promoter region, the iM sequence, and associated transcription factors. The presence of iMs in promoters of oncogenes, tumor suppressors, and other cancer-related genes, as well as in many other genes implicated in metabolism disorders, cardiovascular, autoimmune, and neurological diseases, together with their potential role in gene regulation, make the iM structure also a potential drug target [13,107]. Moreover, based on the fact that the formation of G4 and iM are prevalent in different phases of the cell cycle, it was speculated that they might be mutually exclusive, and they even might play opposite roles in gene expression regulation, although this remain arguable [106]. In fact, it was shown that *k-RAS* promoter G4 stabilizer **nitidine** (Figure 4) destabilizes the complementary i-Motif/hairpin hybrid structure, inducing repression of k-RAS expression [108]. Also, the stabilization or destabilization of iMs with small molecules can lead to upregulation or downregulation of gene expression, depending on the promoter [109,110].

#### 1.2.2. G4s in Human-Infecting Parasites and Bacteria

The first observations on the structural variability of G4s were made with telomeric sequences of environmental protozoans, which have the G-rich repeat T_4_G_4_ [52]. Nowadays, the research on the structural features and biological relevance of G4s has expanded to pathogenic protozoa. Due to the health and economic problems caused by malaria in developing countries around the world [111], one of the most studied genomes, is that of the malaria parasite, *Plasmodium* sp., despite the low G/C-content of this genome. Among the species infecting humans, *P. falciparum* is the one causing the most severe form of malaria [112]. Several studies have demonstrated the presence of G4s-forming sequences in telomeres and in the upstream region of group B *var* genes of *P. falciparum*, a set of genes encoding for a protein that has a significant role in parasite pathogenesis and immune evasion [52,53,113]. Thus, the potential of targeting G4s in *P. falciparum* with small molecules as a strategy to develop new antimalarial drugs has been investigated. The reported activity of G4 ligands against *P. falciparum* in blood cultures is shown in Table 2.

**Table 2:** Antiparasitic and antibacterial activity of G4 ligands. Structures are depicted in Figure 6.

	IC_50_/µM				
G4 Ligand	*P. falciparum*	*T. brucei*	*L. major*	*M. tuberculosis*	Human Cells
**TMPyP4**	35 [114]; 0.62 [51]	>10 [115]	21 [115]	6.25 [116]	>25 [115]
**TMPyP2**	11 [51]				
**Telomestatin**	5 [114]				
**3AQN**	1.8–2.5 [117]				170 [117]
**6AQN**	1.5–2.7 [117]				170 [117]
**PDC-360A**	0.9–1.2 [117]				120 [117]
**Carb-NDI6**	0.275 [115]	0.017 [115]	0.537 [115]		0.71 [115]
**Pyridostatin**	2.65 [115]; 5.2 [53]	7.82 [115]	5.00 [115]		5.38 [115]
**BRACO-19 ^(a)^**	9.70 [115]	5.51 [115]	12.7 [115]	<12.5 [118]	8.33 [115]
**Quarfloxin ^(a)^**	0.11 [51]				
**c-exNDI 2**				<2.5 [118]	
**PIPER-d3**		0.027 [119]	>100 [119]		2.5–53 [119]

^(a)^ Structure in Figure 4.

The initial studies using well-known potent G4-stabilizers and targeting the telomeric G4 of the parasite, which has a G-rich repeat motif slightly different from that of the human telomere, were quite disappointing. All tested molecules, including the trisubstituted acridine **BRACO-19** (Figure 4), the cationic porphyrin **TMPyP4**, the bis-quinolinum **Phen-DC3**, and the macrocycle **telomestatin** (Figure 6) were unable to discriminate between the *Plasmodium* and the human G4s [120]; the capacity to stabilize the G4 was not correlated with inhibition of telomerase activity by **TMPyP4** and **telomestatin**, neither this activity with the inhibition of parasite growth [114].

In addition, **quarfloxin** (Figure 4) showed very good activity against cultured blood-stage parasites. However, subsequent studies on its mode of action could not directly link its antiplasmodial activity to its known high capacity to stabilize G4s [51]. In this study, **quarfloxin** was shown to repress in vitro the transcription of a G4-containing reporter gene in *P. falciparum* (as also did the weak G4-ligand **TMPyP2** in the same assay), but did not affect telomere maintenance, nor disrupted the transcription of rRNAs, the proposed mode of action of **quarfloxin** in cancer cells [98] and trypanosomes [121]. However, recent studies showed more promising results. The bis-quinoline derivatives **360A, 3AQN,** and **6AQN** (Figure 6) were shown to inhibit the growth of intra-erythrocytic parasites with very low toxicity to human cells; to shorten parasite telomeres and activate the transcription of G4-containing subtelomeric genes, such as the *var* genes and others involved in DNA repair and recombination [117]. Moreover, transcriptional profiling of **pyridostatin** (Figure 6) treated parasites showed deregulation of several genes, including those encoding for transcriptional factors and some others involved in ribosome biogenesis [53], thus highlighting the potential of G4s as targets for antimalarial drugs.

Other G4 ligands with in-vitro anticancer activity were also investigated for their potential as new antiparasitic agents against *Trypanossoma brucei* and *Leishmania major*, two other parasites responsible for neglected life-threatening diseases (Table 2). The carbohydrate conjugated naphthalene diimide **carb-NDI6** (Figure 6) showed high activity and selectivity to *T. brucei*. Confocal microscopy confirmed the presence of the compound in the nucleus and kinetoplast of the parasite, where it can target the G4s [115].

Bioinformatic genome-wide analysis revealed the presence of G4-forming sequences in cis-regulatory regions of genes of *Escherichia coli* [122,123,124], *Deinococcus radiodurans* [125], *Xanthomas* sp., *Nostoc* sp., [126] *Streptococcus pneumoniae* [46] and *Mycobacterium tuberculosis* [116,118]. However, a recent whole-genome experimental mapping of DNA G4s in 12 species revealed that compared to eukaryote genomes, bacteria and yeast are depleted of G4s [56]. The role of G4s in controlling gene expression in bacteria has been investigated using different approaches, including gene reporter assays and G4-ligands, as recently reviewed elsewhere [45,127]. These studies show that, like in human cells, gene expression can be up-regulated or down-regulated by G4-stabilizing molecules. For example, it was recently reported that the extended naphthalene diimide **NDI-10** (Figure 6) seems to promote transcription in Gram-positive bacteria. In contrast, it represses transcription in Gram-negative bacteria, probably due to differences in G4 DNA prevalence between the two groups [128]. Among the most complete and interesting studies are those identifying G4 motifs as responsible for antigenic variations in *Neisseria gonorrhoeae* [129,130] and in genes of *Mycobacterium tuberculosis* responsible for virulence and survival of bacteria inside the host cell [116]. Moreover, G4 ligands were shown to inhibit the growth of *M. tuberculosis* inside host cells at microM concentrations (Table 2) and inhibit the transcription of identified G4-containing virulence genes, thus demonstrating the potential of G4s as therapeutic targets for the development of new anti-tuberculosis agents.

#### 1.2.3. G4s in Human Viruses

Targeting G4 structures in DNA or RNA viral genome for antiviral therapy is still underexplored. Putative G4-forming sequences have been detected in the genome of all human viruses, including those involved in recent epidemics and pandemics, such as the Ebola, Zika, influenza H1N1, and SARS-CoV-2 viruses [131,132,133,134]. DNA viruses, such as herpesviruses, have been found to be particularly enriched in G4s compared to RNA viruses [131]. Moreover, most characterized viral G4s are in conserved genomic regions, an important aspect if we want to consider them as drug targets since viruses are characterized by high mutation rates [135]. Table 3 summarizes the main findings up to date on the antiviral activity and mechanism of action of known G4-stabilizers. Details on the viral life cycles and positions and roles of G4s in each viral genome can be found in another recent review [136]. The porphyrin **TMPyP4** (Figure 6) and the acridine derivative **BRACO-19** (Figure 4) are the two G4 ligands most used to study the role of G4s in viral life cycles and the potential of G4s as drug targets, whereas the DNA genome of Herpes Simplex Virus 1 (HSV-1) and the RNA genome of Human Immunodeficiency Virus 1 (HIV-1) are the two most investigated for the potential of G4s as therapeutic targets. Results from the studies summarized in Table 3 show highly promising results.

**Figure 7:**
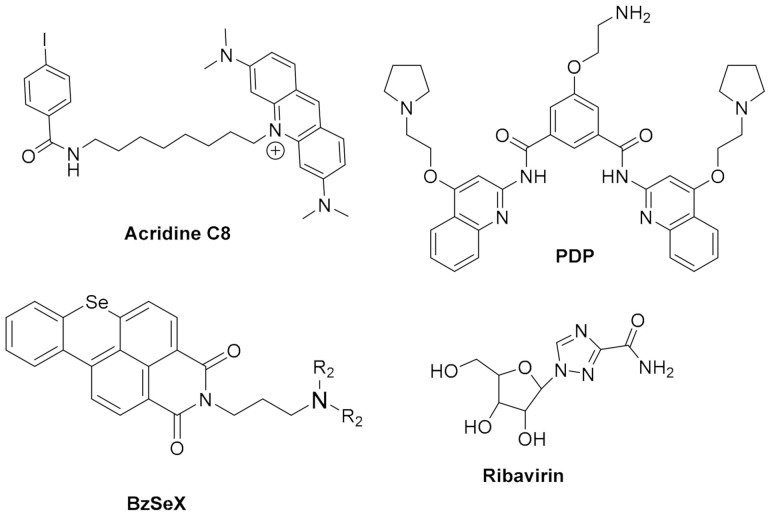
Structures of other G4 ligands with antiviral activity.

Most small molecules that could stabilize in-vitro the viral G4 structures also showed good antiviral activities and the capacity to interfere with viral replication and latency. Considering that current antiviral therapies are unable to target latent viruses, such as HIV and human herpes viruses, targeting the viral G4s may represent an exciting new pharmacological approach to eradicate these viruses. However, it must be noted that not all G4-stabilizers induce the same effect. For example, **pyridostatin** (Figure 6) stabilized the G4 in mRNA of the Epstein–Barr Virus (EBV) encoded Nucleic Antigen 1 (EBNA1), inhibiting its translation but **PhenDC3** (Figure 6) in the same study, showed no effect on EBNA1 translation [137]. Moreover, another study showed that most G4s in the Human Cytomegalovirus (CMV) gene regulatory regions form stable G4s. Still, experiments using reporter assays and G4-stabilizer small molecules show that only a few G4s suppress viral gene expression [138]. Another promising approach is to induce an immune response by targeting the interaction protein-G4. **PhenDC3** was found to block cellular nucleolin binding to EBNA1 mRNA G4s, leading to increased EBNA1 levels in EBV-infected cells and consequent antigen presentation [139,140]. Given the recent pandemic caused by the new RNA virus called Severe Acute Respiratory Syndrome Coronavirus-2 (SARS-CoV-2), there is an urgent need for a therapeutic agent. The genome and transcriptome of this virus have been extensively investigated to find suitable drug targets. G4-forming sequences have been predicted to occur in the open reading frames (ORF) and coding sequence (CDS) regions of nucleocapsid protein (N) and spike glycoprotein (S), in both positive-sense and negative-sense RNA strands of SARS-CoV-2. The most conserved sequences, among beta coronaviruses, were also confirmed to form G4s in-vitro and to be unfold by viral helicases and host Cellular Nucleic Acid Binding Protein (CNBP) [133,141,142,143,144,145]. Recent in-vitro studies show that G4 ligands, such as **TMPyP4** and **BRACO-19,** can repress SARS-CoV-2 G4-base gene expression [145] and others like **PhenDC3**, **PDC-360A**, or metalated porphyrins, at nanomolar concentrations, can disrupt the interaction between RNA G4 and the SARS-Unique Domain (SUD) of Nsp3-protein, an essential protein for viral replication [146]. Also, the G4-stabilizer **PDP** (Figure 7) was shown to stabilize the G4 in the CDS of N and inhibit its translation, both in-vitro and in-vivo at 2-4 microM concentrations, with consequent reduction of the protein levels of SARS-CoV-2 N [147].

Another potential target is the G4-forming sequence in the promoter of the human transmembrane serine protease TMPRSS2, which has been shown to play an important role in SARS-CoV-2 and Influenza A virus (IAV) entry into the host cells [148,149,150]. Benzoselenoxanthene derivatives (e.g., **BzSeX**; Figure 7) were shown to be in-vitro good stabilizers of the G4 structure in the TMPRSS2 promoter and to decrease the protease expression and viral replication [151]. Moreover, **ribavirin**, a known antiviral drug (Figure 7) with in-vitro activity against SARS-CoV-2, was shown to decrease the expression of TMPRSS2, at both mRNA and protein levels, as well as the AC2 expression and TMPRSS2 enzymatic activity [152]. Although **ribavirin** is not expected to be a G4 ligand, it is able to inhibit the activity of helicases by competitive inhibition of ATP hydrolysis [153], which would lead to an indirect stabilization of G4s.

**Table 3:** Antiviral activity of selected G4 ligands.

G4 Ligand (Chemotype)	Structure in Figure	Virus	Effects	Refs.
**TMPyP4** (porhyrine)	6	HIV-1	Blocked viral replication in lymphocyte T cells with established HIV-1 latency.	[154]
			Inhibition of viral infectivity.	[155]
			Enhanced killing of latently infected cells when in combination with latency reversing agents.	[156]
		KSHV	Inhibited viral DNA replication; reduced in 60% the viral episome copy numbers; inhibited LANA1 translation in KSHV infected cells.	[157,158]
		HCV	Promoted viral RNA polymerase stalling.	[159]
		EBOV	Reduced transcription of L gene (encodes for viral RNA-dependent RNA polymerase) and impaired replication of viral genome.	[160]
		HSV-1	Showed good antiviral activity at microM concentrations; did not inhibited virus DNA replication or entry but inhibited virus release by the cells.	[161]
		ZIKV	Inhibited viral growth, genome replication and protein expression.	[162]
		SARS-CoV-2	Inhibited replication and gene expression of virus RNA G4-forming sequences in in-vitro assays	[145]
**Acridine C8**	7	HPV	The exposure of cervical cells to C8 at 0.25 microM induced a >100-fold decrease in HPV18 viral titre; C8 probably affects viral genome encapsidation rather than genome amplification.	[163]
**BRACO-19** (acridine)	4	HIV-1	Reduced viral titre to undetectable levels in latently infected cells.	[154]
			Blocked RT progression in-vitro, which was counteract by viral Ncp7, a protein known to unfold RNA G4s.	[164,165]
			Antiviral activity at microM concentrations.	[164]
			Reduced proviral LTR promoter activity.	[165]
			Enhanced killing of latently infected cells when in combination with latency reversing agents.	[156]
		HSV-1	Antiviral activity (IC_50_~8 µM) with inhibition of viral DNA synthesis.	[166]
		HHV-6	Reduction of viral genome integration in human chromosomes at telomeres.	[167]
		EBV	Reduced viral genome copy numbers in infected lymphocytes; reduced transcription of viral proteins EBNA2 and EBNA3A; reduced EBNA1-dependent DNA replication.	[168]
		HBV	Enhanced preS2/S gene promoter activity, which product regulates production of the HBV surface antigen and virion secretion.	[169]
		ZIKV	Inhibited viral growth, genome replication and protein expression.	[162]
		SARS-CoV-2	Inhibited replication and gene expression of virus RNA G4-forming sequences in in-vitro assays	[145]
**c-exNDI** (naphetalene Diimide)	6	HIV-1	Strong antiviral activity (IC_50_ < 25 nM).	[170]
		HSV-1	Antiviral activity (IC_50_~18 nM) with inhibition of viral DNA synthesis.	[171]
**Pyridostatin**	6	EBV	Reduced EBNA1 synthesis and recognition of EBV-infected cells by virus-specific T cells.	[137]
		HBV	Enhanced preS2/S gene promoter activity.	[169]
		ZIKV	Inhibited mRNA synthesis, virus cytopathic effect and viral NS2B-NS3 protease activity in infected Vero cells, particularly during postinfection treatment.	[172]
**PDP** (pyridostatin)	7	HCV	Promoted viral RNA polymerase stalling. In-vivo G4-mediated antiviral activity in the low microM range.	[159]
		SARS- CoV-2	Inhibited translation of nucleocapsid protein N, in-vitro and in-vivo.	[147]
**CX-5461** (quinolone)	4	CMV	Reduced viral titre by 2 log, acting at the viral DNA replication stage.	[173]
**PhenDC3** (phenanthroline)	6	EBV	Inhibited nucleolin binding to EBNA1 mRNA G4s and increased the endogenous EBNA1 levels in EBV-infected cells.	[139]
		KSHV	Inhibited viral DNA replication by stalling the replication fork at the TR level; consequent reduction of viral episome copy numbers.	[157]
		HCV	Inhibited viral replication in cells.	[174]
		SARS-CoV-2	Inhibited in-vitro the SUD-NM/TRF2 G4 interaction with an IC_50_ = 51 nM.	[146]
**BzSeX** (Benzoseleno xanthene)	7	IAV	Reduced viral titers in-vitro, with downregulation of TMPRSS2 expression, a transmembrane serine protease essential for virus entry into the host cells	[156]
**ribavirin**	7	SARS-CoV-2	Antiviral activity; reduced expression of TMPRSS2 and AC2; inhibited TMPRSS2 enzymatic activity.	[152]

dsDNA viruses: HSV-1 Herpes Simplex Virus 1; HHV-6 Human Herpesviruses 6A/6B; KSHV Kaposi’s Sarcoma-associated Herpes virus; HPV Human Papilloma Virus; EBV Epstein–Barr Virus; CMV Human cytomegalovirus; HBV Hepatitis B Virus. (+)ssRNA viruses: HIV-1 Human Immunodeficiency Virus 1; HCV Hepatitis C Virus; EBOV Ebola Virus; SARS-CoV-2 Severe Acute Respiratory Syndrome Coronavirus-2; ZIKV Zika Virus; IAV Influenza A virus. Legend: EBNA: EBV-Encoded Nuclear Antigen; LANA1: Latency Associated Nuclear Antigen 1, a regulatory protein of virus latency; LTR: Long terminal Repeats; RT: Reverse Transcriptase; TR: Terminal Repeats (a guanine rich region).

### 1.3. Approaches in the Design of Quadruplex-Interactive Small Molecules

The search for quadruplex-interactive small molecules has followed the classical medicinal chemistry strategies, like the rational design of lead molecules and their optimization or the use of high throughput techniques to identify new lead molecules.

**Figure 8:**
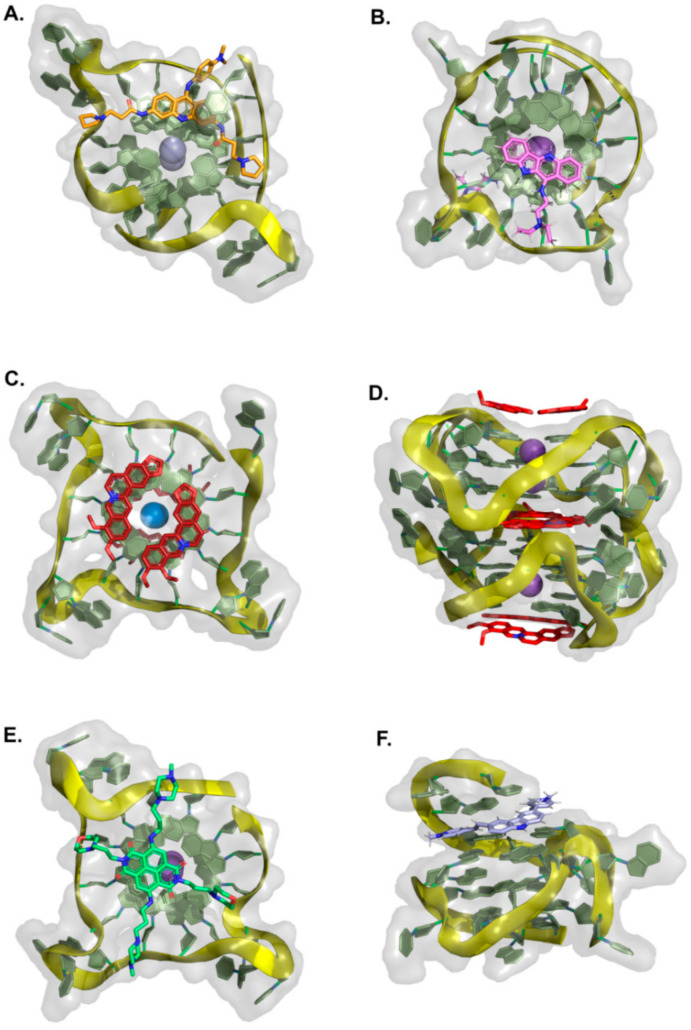
Structural representation of different classes of fused aromatic polycyclic ligands (colored sticks) in complex with different G4s (represented as cartoon and transparent surface). In (**A**) we have represented BRACO-19 in complex with a parallel human telomeric G4 (3CE5); in (**B**) a indoloquinoline derivative in complex with a *c-MYC* G4 (2L7V); in (**C**,**D**) berberines in complex with a human telomeric G4 (3R6R—[206]); in (**E**) the complex of the naphthalene diamine MM41 and human telomeric DNA G4 (3UYH) and in (**F**) the representation of the BMVC carbazole interacting with a parallel *c-MYC* promoter DNA G4 (6JJ0).

The rational design of G4-interactive small molecules based on G4 structure has led to molecules with large flat aromatic polycyclic systems, able to stack on the external G-quartets (Figure 8) but unable to intercalate into DNA double-helix. Optimization of these scaffolds towards stronger G4 stabilizers usually implies the addition of one or two side chains with pH-dependent protonable groups [86,175,176,177]. In an attempt to increase both drug-like properties and target binding specificity of G4 ligands, many other more flexible and smaller molecules were developed [178]. In many cases, in-silico techniques using G4 structures determined by X-ray crystallography or NMR, have been used to design new molecules or withdraw structure-binding relationships for future studies [179,180,181,182,183]. In this regard, Neidle has recently pointed out that these studies consider only the isolated G4 structures, which does not reflect the cellular environment in which G4s are embedded with duplex DNA, folded mRNA, or chromatin contexts [184]. Moreover, special attention must be paid when choosing the G4 structure to be used in computer-assisted design of G4-interactive molecules since clusters of structured water molecules play essential roles in mediating the interaction between ligand side chains groups chromophore core and G-quadruplex [185].

Virtual and in-vitro screening campaigns to identify new chemotypes have also been pursued. Docking and pharmacophore-based methods are the most commonly adopted virtual screening strategies, and several examples of new G4-interactive small molecules identified by these methods, as well as their strengths and caveats, were recently reviewed [186]. In-vitro screening campaigns usually use a FRET melting assay, one or more G4-forming sequences, as well as chemically diverse large compound libraries [187,188] or more focused ones, such as those of NCI [189]. More recently, custom G4 microarrays have been developed to assess the binding selectivity of G4-interactive small molecules and to help understand the chemical features that govern molecular recognition [190,191].

## 2. G4-Ligand Complexes and Intermolecular Interaction Modes

This section is intended not to catalog all the interaction complexes between G4s and the small molecules described in publications throughout the past years but to highlight the main classes and interaction motifs of representative organic compounds with proven affinity, available at the RCSB Protein Data Bank [192]. This section will specifically explore the main structural features that regulate the interaction of the three major classes of organic G4 ligands: fused polycyclic ligands, modular ligands, and macrocycles.

### 2.1. Fused Polycyclic Ligands

**Figure 9:**
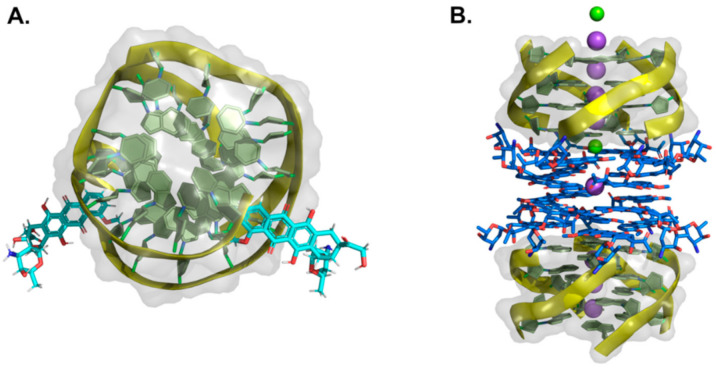
Structural representation of the human telomeric G4 (cartoon and transparent surface) in complex with (**A**) adriamycin (6KN4) and (**B**) daunomycin (3TVB) (represented as colored sticks).

**Figure 10:**
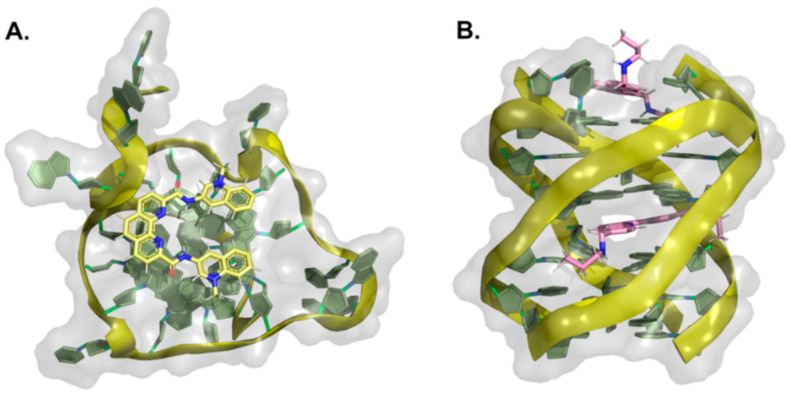
Representation of (**A**) a phenanthroline derivative in complex with G4 derived from the *c-MYC* promoter (2MGN) and (**B**) a quinacridine derivative in complex with a tetramolecular G4 (2JWQ).

Fused polycyclic G4 ligands are characterized by having a scaffold core moiety composed of fused aromatic rings. Such class of compounds was explicitly developed to have a planar shape with affinity to the terminal 3′ and 5′ G-quartets, coupled to tetra, bi, or unimolecular cationic pendants, for further electrostatic stabilizing interactions with the G4 phosphate backbone regions. In the following sub-sections, we will go through representative examples of complexes between G4 and acridines, indoloquinolines, berberines, anthracyclines, naphthalene diimides, phenanthrolines, quinacrines, and carbazole derivatives (Table 4 and Figure 8, Figure 9 and Figure 10).

#### 2.1.1. Acridines

In recent years, tricyclic acridine-containing compounds have been actively investigated as small chemotherapeutic anticancer agents [193]. These compounds were shown to be potent inhibitors of topoisomerase and telomerase function during replication of the cells, which ultimately leads to apoptosis and cell death (see Table 1). The planar heteroaromatic chromophore characteristic of these compounds establishes strong π–π interactions with the G-quartet motif at the terminus of a G4, with short alkyl chain substituents with protonated amino groups (at physiological pH) found to determine its affinity and selectivity [194,195]. The presence of a protonated nitrogen atom at physiological pH in the heterocyclic scaffold increases the electron deficiency in the chromophore, with consequent enhancement of the G4 interaction. Additionally, the length and the nature of the bi and tri side chains terminal groups, many functionalized with tertiary amine moieties fully protonated at physiological pH, boost the affinity and the binding energetics to the DNA and RNA G4 grooves (Figure 8A). Interestingly, several different structures found at the RCSB databank [192], evidence that these cationic groups do not directly interact with the G4 phosphates, but instead with bridging water molecules found intercalating the pairs of bases in the flexible grooves, which were shown to play important roles in G4 ligand interactions [185].

Different acridines were resolved complexed with both DNA and RNA G4s (Table 4). In the case of RNA-based G4 (e.g., 3MIJ [196]), it is possible to identify a larger available area of interaction with the ligands when compared to DNA G4s. This difference has an evident impact on the number of acridine molecules each RNA G4 can accommodate. Typically, while in the RNA G4 complexes, one can observe the existence of two acridines binding to the G-quartet terminal region, in DNA G4s, the available surface only allows the binding of one molecule.

#### 2.1.2. Indoloquinolines

Another example of efficient fused aromatic polycyclic ligands binding to G4s is the indolo[3,2-*b*]quinoline or quindoline derivatives. These four aromatic-ring-based compounds were initially described to interact with double-strand DNA through intercalation. Still, with the publication of the 2L7V NMR structure (Figure 8B) [197] of an indoloquinoline derivative interacting with the parallel-stranded *c-MYC* promoter G4, it was possible to see that this type of compounds interacts with their aromatic chromophore at the terminal G-quartet of the G4s through a typical π–π stacking interaction.

Interestingly, in this structure, it is possible to identify two indoloquinoline molecules binding the G4 at both its 3′ and 5′ ends, directly over the external G-quartets. Although minor differences in the interaction of these two compounds can be observed (most due to differences in the base pair sequences in each terminal of the G4), the binding of the indoloquinoline to the 5′-end seems to be more favored since this region of the G4 is more hydrophobic and accessible for ligand stacking. Notwithstanding, each indoloquioline molecule binds to the external quartets in an “induced-fit” arrangement, where flanking segments changed dramatically from their free state to allow the formation of a favorable ligand binding arrangement, with the indoloquinolines stacking over a total of three of the four guanines in the external G-quartets, and with flanking residues wrapping over each indoloquinoline-base planes. Furthermore, it is also possible to observe that the amine group in the side chain attached to the indoloquinoline core additionally contributes to a stable interaction due to favorable electrostatic interactions with the phosphate groups of the G4 backbone, similarly to what is observed in other classes of compounds.

#### 2.1.3. Berberines

Isoquinoline alkaloids, such as berberines, constitute a class of natural products commonly used in different variants of folk medicine [198]. This sub-class of fused aromatic polycycle G4 interacting compounds possesses striking biological and pharmacological features, which consequently have been seen as promising emergent therapeutics in contemporary biomedical research [199,200]. In recent years these compounds have been reported to bind with high affinity to G4 arrangements, illustrating again that the properties and behavior of these compounds are becoming a key area of study and interest [201,202,203,204,205]. Furthermore, the synthetic 9-O substituted berberine derivatives show an even higher specificity and binding affinity when compared to the reference berberine, as will be discussed next.

In the RCSB databank, several berberine derivatives are found mainly in complex with human telomeric G4s (Table 4). For example, in the structure with reference 3R6R (Figure 8C) [206], one can identify an intramolecular G4 in complex with berberine in a molar ratio of DNA/ligand 1:4. The observed DNA topology is of the parallel type, and as can be seen in Figure 8C, none of the berberine molecules are found close to the G4 loops. Instead, the packing of this crystal (Figure 8D) evidence two symmetric G4 dimers featuring an interaction of the two 5′-ends (of each symmetric unit), which define a binding site where two coplanar berberine molecules are stacked. Each of these molecules interacts with a pair of adjacent guanines per G-quartet. Due to the twofold symmetry of the binding site, each ligand interacts with the same couple of guanines from the two different G4 quartets.

Recently [207], the X-ray structure (PDB reference—6S15) of a new berberine derivative, with a feature pyridine side group at the 13′ position, was resolved together with a human telomeric DNA sequence forming two consecutive G4s.

In all the evaluated arrangements, only one ligand was found sandwiched between the two G4 symmetric units, in contact with the 3′-end G-quartet from one unit and the 5′-end G-quartet from the other symmetric G4 unit. Interestingly, the alkaloid core interacts with the G-quartet pointing its cationic nitrogen away from the center, giving rise to a π−π stacking with two guanines. The aromatic pendant at the 13′ position also faces away from the central core of the interacting G-tetrad, consistently facing crystallographic water molecules.

#### 2.1.4. Anthracyclines

Anthracyclines are a class of compounds commonly used in cancer chemotherapy to treat several types of cancers, including leukemia and lymphomas [208], albeit in association with cumulative dose-associated cardiotoxicities. These compounds are extracted from *Streptomyces* spp. and have been characterized as efficient inhibitors of DNA and RNA synthesis and topoisomerase II [209]. Several derivatives of this class of compounds, such as daunorubicin, doxorubicin, and epirubicin, are anticancer agents in clinical use. They are aromatic planar molecules consisting of a rigid hydrophobic tetracyclic system, with an amino sugar attached through a glycosidic bond. Several reports have shown that anthracyclines can interact with telomeric DNA and may bind and stabilize telomeric G4 [210,211,212]. Currently, at the RCSB databank, it is possible to find several NMR and X-ray structures of this class of compounds in complex with G4 arrangements (Table 4). In the most recent structures, we can find adriamycin (PDB code 6KN4—Figure 9A) [213] and epirubicin (PDB code 6KXZ) [214], not having a planar stabilizing and direct interaction with G4 G-quartets as observed with other fused aromatic polycyclic ligands. In these complexes, even though both ligand molecules present a partial binding of their anthraquinone ring with the G4, the position of the daunosamine sugar moiety is found in the opposite direction.

In the epirubicin complex reported in the structure with PDB code 6KXY, this chemical group interacts directly with the 5′ of the G4. In contrast, in the adriamycin complex (6KN4–Figure 9A), this group is observed to be in an opposite position to the 5′ end of the G4, with an even higher degree of solvent exposure. Interestingly, the topology of interaction observed for these two anthracyclines is entirely distinct from the conformations observed in the structures where daunomycin is complexed with a d(GGGG)4 (3TVB [215]) and a non-human telomeric G4 (1O0K [211]). In these structures (Figure 9B), pairs of sandwiched layers of daunomycin molecules are tightly stacked through van der Waals interactions between the two units of the G4s. Interestingly, despite the distinct G4 DNA geometries, a similar high surface area of interaction with the daunomycin molecules is observed. In the 3TVB structure, the DNA adopts an unusual conformation with unique interlayer guanine rotations and no daunosamine groove insertions from the daunomycin layer structures. However, in the 1O0K structure [211], while the daunomycin molecules layer pack tightly onto the end of the quadruplex stack, the daunosamine sugar moieties establish hydrogen bond interactions and/or van der Waals contacts with three of the four quadruplex grooves.

#### 2.1.5. Naphthalene Diimides

The naphthalene diimide (NDI) core [216] is the smallest homolog of the rylenediimides [217], with high electron affinity, good charge carrier mobility, and excellent thermal and oxidative stability, characteristics that provide good water solubility, permeability, pharmacokinetic, and toxicity profiles [218,219]. As mentioned in the previous section, this class of compounds is among the most promising anticancer G4 targeting drugs due to their ability to simultaneously target multiple G4s and their strong and selective anticancer activity [220]. Indeed, their proven ability to interact with G4 quartets and their chemical accessibility and the possibility to easily functionalize their aromatic cores with multiple, diverse pendant groups allow for a fine modulation of their chemical structure providing a solution to address possible toxicity and DNA selectivity issues.

Several examples of NDIs complexed to human G4s can easily be accessed through PDB references 3SC8 [221], 3UYH (Figure 8E) [179], 3T5E [221], 4DAQ [179], 4DA3 [179] (Table 4). In each one of these structures, we can find several examples of tetra-substituted NDI derivatives with positively charged termini, which are potent stabilizers of human telomeric DNA G4s. This class of molecules typically stack over the terminal G-quartets binding surfaces, with extensive π−π contacts, in a 1:1 ligand/G4 stoichiometry. This type of stoichiometry is different from what is observed in other PDB reference structures such as 3CCO [222] and 3CDM [222], where the ligands (phenanthrolines) with two different substituent group pairs display multiple binding modes with the telomeric G4s. Focusing specifically on the 3SC8 and 3T5E structures resolved by Collie et al. [221], the NDI compounds BMSG-SH-3 and BMSG-SH-4 bind to parallel-stranded G4 topology, with three characteristic chain-reversal propeller type loops. Both NDI complexes involve two individual G4 units stacking at the 5′ G-quartet interface in a process mediated by a coordinating potassium ion. In these structures, many of the NDI side-chains are placed within the G4s groove regions, interacting through different hydrogen bonds, water bridges, and electrostatic contacts with the negatively charged phosphate groups, as previously reported for other branched fused polycyclic aromatic ligands [185].

#### 2.1.6. Phenanthrolines and Quinacridines

Another class of compounds known to intercalate the base pairs of DNA and produce cytotoxic effects against tumoral cells are phenantrolines and quinacridines [223,224]. These two classes of compounds were additionally described to stabilize G4 arrangements, and currently, at the RCSB databank, one can find several structures of such compounds in complex with G4s: 2MGN [225] (Figure 10A) and 2JWQ [226] (Figure 10B).

**Table 4:** Summary table with some reference complex structures of quadruplexes with fused aromatic polycyclic ligands available at the RCSB protein databank.

PDB ID	Method	Ligand Chemotype	Quadruplex	Refs.
**1K2L**	X-ray	Acridine	Four-way DNA junctions	[227]
**1NZM**	NMR	Acridine	Parallel hTelo DNA G4	[228]
**1L1H**	X-ray	Acridine	Antiparallel DNA G4 from *Oxytricha nova*	[210]
**3CE5**	X-ray	Acridine (BRACO-19)	Parallel hTelo DNA G4	[229]
**3EUM**	X-ray	Acridine	*Oxytricha nova* bimolecular G4	[230]
**3EUI**	X-ray	Acridine	*Oxytricha nova* bimolecular G4	[230]
**3EM2**	X-ray	Acridine	*Oxytricha nova* bimolecular G4	[230]
**3EQW**	X-ray	Acridine	*Oxytricha nova* bimolecular G4	[230]
**ERU**	X-ray	Acridine	*Oxytricha nova* bimolecular G4	[230]
**3ES0**	X-ray	Acridine	*Oxytricha nova* bimolecular G4	[230]
**3ET8**	X-ray	Acridine	*Oxytricha nova* bimolecular G4	[230]
**3NZ7**	X-ray	Acridine	Antiparallel DNA G4 from *Oxytricha nova*	[231]
**3NYP**	X-ray	Acridine	Antiparallel DNA G4 from *Oxytricha nova*	[231]
**3MIJ**	X-ray	Acridine	Parallel hTelo RNA (TERRA)	[196]
**3QCR**	X-ray	Acridine	Parallel hTelo RNA (TERRA)	[196]
**5LIG**	NMR	Acridine	c-Myc promoter parallel DNA G4	[232]
**2L7V**	NMR	indoloquinoline	c-Myc promoter parallel DNA G4	[197]
**3R6R**	X-ray	Berberine	Parallel hTelo DNA G4	[206]
**4P1D**	X-ray	Berberine	Bimolecular hTelo DNA G4	[233]
**5CDB**	X-ray	Berberine	Antiparallel hTelo DNA G4	[234]
**6CCW**	NMR	Berberine	Hybrid hTelo DNA G4	[235]
**6S15**	X-ray	Berberine	Bimolecular hTelo DNA G4	[207]
**6JWD**	NMR	Berberine	RET promoter parallel DNA G4	[236]
**1O0K**	X-ray	Anthracyclin	Intermolecular parallel Telo DNA G4	[211]
**3TVB**	X-ray	Anthracyclin	Parallel Telo DNA G4	[215]
**6FC9**	NMR	Anthracyclin	Quadruplex-duplex junction	[237]
**6KXZ**	NMR	Anthracyclin	Parallel hTelo DNA G4	[214]
**6KN4**	NMR	Anthracyclin	Parallel hTelo DNA G4	[213]
**3SC8**	X-ray	Naphthalene diimide	Parallel hTelo DNA G4	[221]
**3T5E**	X-ray	Naphthalene diimide	Parallel hTelo DNA G4	[221]
**3UYH**	X-ray	Naphthalene diimide	Parallel hTelo DNA G4	[179]
**4DA3**	X-ray	Naphthalene diimide	Parallel hTelo DNA G4	[179]
**4DAQ**	X-ray	Naphthalene diimide	Parallel hTelo DNA G4	[179]
**3CDM**	X-ray	Phenantroline	Parallel hTelo DNA G4	[222]
**3CCO**	X-ray	Phenantroline	Parallel hTelo DNA G4	[222]
**2MGN**	NMR	Phenantroline (PhenDC-3)	c-Myc promoter parallel DNA G4	[225]
**2JWQ**	NMR	Quinacridine	Tetramolecular G4 derived from hTelo	[226]
**6JJ0**	NMR	Carbazole	c-Myc promoter parallel DNA G4	[238]
**6O2L**	NMR	Carbazole	c-Myc promoter parallel DNA G4	[238]

hTelo: human telomere.

While in the 2MGN structure, the central part of the ligand (**Phen-DC3**, Figure 6) is a typical phenanthroline moiety, in the structure with reference 2JWQ, the central moiety of the ligand is a derivative of a phenanthroline, also categorized in the acridines’ class of compounds, named as quinacridines. Overall, one can observe in structure with reference 2MGN that the phenanthroline moiety establishes optimal interactions with the top G-quartet, with the quinolinium groups placed directly away from the more flexible 5′ end, facing the propeller groups, assuring a minimum number of steric clashes with the more flexible regions of the quartets. The two N-methyl groups are found above the grooves, conserving minimal contact with the quadruplex surface but assuring the aromatic complementarity with the G4 surface. Evidence shows that when the quinolinium groups are substituted by pyridium, a negative impact on the thermal stability of the reported complexes was obtained [239]. If we compared this interaction topology with the one observed between the tetramolecular quadruplex from [d(T_2_AG_3_T)]_4_ with a quinacridine (PDB reference 2JWQ), it is clear the existence of 2 molecules:1 G4 binding stoichiometry. According to the reported work [226], one of the ligands stacks on the top of the tetramolecular G4, stabilized by hydrophobic π−π stacking between the quinacridine moiety and the top G-quartet with the two cationic branches binding to the flanking grooves. The second quinacridine molecule intercalates the G4 arrangement. Its quinacridine moiety is sandwiched between the G-tetrad formed by G4, G11, G18, and G25 and the A-tetrad formed by A3, A10, A17, and A24, with both branches of the compound, being well accommodated along two grooves of the G4.

#### 2.1.7. Carbazoles

In 2019, the 3D structure of a carbazole derivative was solved in two different NMR structures, showing a 1:1 (PDB code 6JJ0, Figure 8F [238]) and a 2:1 (PDB code 6O2L [238]) chemical ratio of interaction with c-MYC DNA G4′s. This compound, also known as BMVC, was initially described as a G-quadruplex-specific ligand and the first in-vivo fluorescent probe to detect the G-quadruplex structures in human telomeres [240,241,242]. Furthermore, BMVC was afterward shown to be a promising fluorescent marker of cancer cells and a potential antitumor agent [243,244,245]. Liu et al. [238] reported that in both the 1:1 and 2:1 BMVC-G4 ratio complexes, the compound perfectly stacked its central carbazole and the two-branched pyridine groups with three bases of the terminal G-quartet with an optimal π–π stacking. Notably, it was also reported that the rotatable double-bond linker of the BMVC molecule enables it to adjust its conformation, varying between extended and the more stable contracted conformation. This highly optimized interaction with G-quartet was afterward validated to be vital for the high-affinity binding of BMVC to c-MYC DNA G4.

### 2.2. Modular Ligands

Here, our focus will be to overview the major structural features regulating the interaction of different G4 topologies with modular G4 ligands. In this class of compounds, the aromatic modules composed of up to two fused rings are combined through different types of bond connections to create flexible structural motifs that ultimately assure stable complexes, with the necessary amount of flexibility to guarantee the desired selectivity for different G4 topological arrangements. Overall, we will divide our analysis into three different subcategories, focusing on modular ligands with aryl–aryl, carboxamide, and olefin-based connections.

#### 2.2.1. Ligands with Aryl–Aryl Bonds

**Figure 11:**
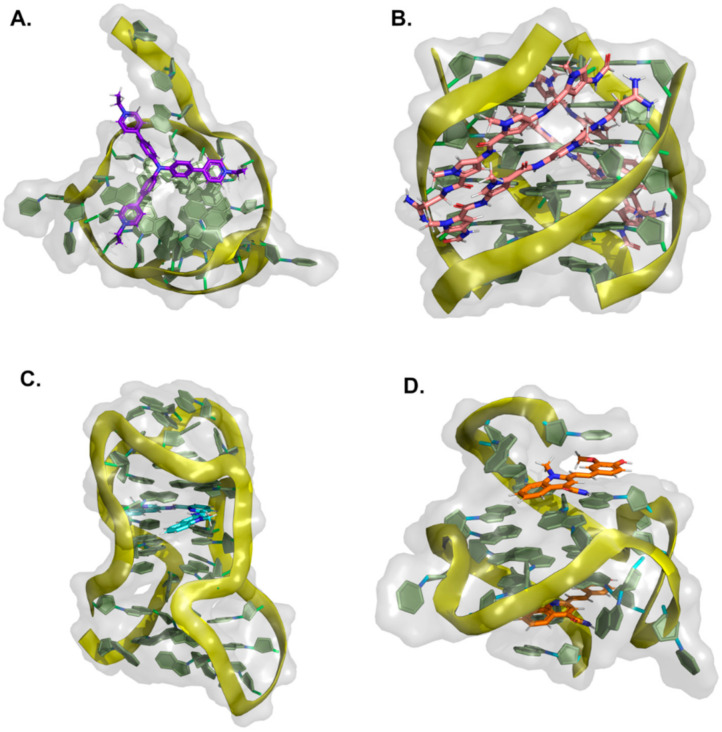
Structural representations of the complexes between G4s (represented as cartoon and transparent surface) and (**A**) a NBTE probe (PDB 6KFJ), (**B**) distamycin A (PDB 2JT7 ]), (**C**) PDC-360A (PDB 6SX3 ]) and (**D**) PEQ (PDB 7KBX) which are represented with coloured sticks.

Aryl–aryl bond connections are found in several compounds known to efficiently bind to G4 (e.g., triarylpyridine **TA20** in Figure 4). However, not so many 3D structures of this type of compound in complex with G4s have been deposited at the protein databank. One of the few available examples of such complexes is found in structures with PDB reference 6KFJ and 6KFI [246] (Table 5). In these structures, one can see the complex between the tripodal cationic fluorescent probe NBTE (a tri-arylamine) and the telomeric DNA G4. As described in this work, upon the NBTE binding to the G4, considerable conformational changes were observed at the 5′-end of the G4s, forming an induced-fit binding pocket for the probe. Interestingly, the NBTE probe is not located within the center of the 5′ G-quartet but shifted toward the edge of the G-quartet and stacked on two guanines, due to the steric hindrance of a lateral loop (Figure 11A). Two arms of NBTE recruit an adenine to form an adenine–NBTE stacking moiety covering the 5′ G-quartet, while the third arm stretches out of the G-quartet. Notably, this arm of NBTE is covered and stabilized by one of the adenine residues of the flanking triad. The binding of NBTE occurs mainly through π–π stacking with the lower 5′ G-quartet and the upper induced capping triad, as well as π–cation interactions and electrostatic interactions at the 5′-end resulting from the positive charge of the three ethylated pyridine groups.

#### 2.2.2. Ligands with Carboxamide Based Linkers

From the compounds with carboxamide-based connections, one must in first place, highlight **distamycin A** and some derivatives shown to bind with high affinity to G4 [247], as well as with duplex DNA. Interestingly, in the two structures available of this compound complexed with DNA G4s (PDB codes 2JT7 [248] and 2KVY [249]), the molecules show a different interaction profile from most of the G4 stabilizers classes, as they are groove binders. In these two reported complexes, distamycin molecules interact with telomeric G4s in a 4:1 binding mode, with two distamycin dimers simultaneously binding two opposite grooves of the quadruplex (see Figure 11B). Analysis of the distamycin-G4 interaction patterns revealed that this ligand binds as an antiparallel dimer to the quadruplex groove by adopting a crescent shape and establishing H-bonds with the G4 bases [248]. Moreover, strong Coulombic interactions are established between the ligand’s positively charged amidinium terminal group and the backbone phosphate groups of the G4.

Additionally, in 2008, Calabrese et al. [181] reported the complex between a c-MYC DNA G4 and the benzofuran derivative **DC-34**. This small molecule, with a distinct chemical profile, when compared to the previously mentioned distamycin, has its aromatic rings connected by a carboxyamide group (PDB code 5W77). According to these authors, this compound targets with high affinity the c-MYC DNA G4 (in comparison with other G4 structures) and ultimately downregulates MYC transcription in cancer cells. This compound’s benzofuran and para-trifluoromethylbenzene rings establish π–π stacking interactions with the G-quartets, while the azepane ring is directed away to the solution. In this structure, a 2:1 complex is formed, with both compounds establishing the previously described type of interaction with the exposed 3′ and 5′ G-quartets of the parallel G4.

The ligand **PDC-360A** (Figure 6) is another example of a compound with carboxamide linkers recently resolved in complex with a G4 (PDB reference 6SX3—[250]). This compound, previously shown to display a strong affinity and selectivity for G4 structures and telomerase inhibition [251], was reported to bind to a VK2 AGCGA tetrahelical quadruplex through intercalation between GAGA and GCGC quartets (Figure 11C). The observed interaction topology is one of the few reports of a heterocyclic ligand with an intercalated configuration in a quadruplex, differently from the previous suggestions presuming this compound would stack on the outer G-quartets of intramolecular G4s [251,252]. In the 6SX3 structure, the small and flexible aromatic scaffold of **PDC-360A** allows the intercalation in the central cavity of VK2, in a configuration stabilized through π–π aromatic interaction and favorable electrostatic interactions between the cationic quinolinium groups and the quadruplex backbone. Interestingly, the reported complex showed no perturbation in its overall structure upon the binding of **PDC-360A**, in contrast to its effect after binding to telomeric G4s [183,253,254]. The size of the ligand regulates the binding into the central cavity of VK2. This information agrees with the data also reported in this work, stating that a significantly lower affinity and much less effective thermal stabilization of VK2 was obtained for the other two bis-quinolinium ligands: **pyridostatin** and **Phen-DC3**. These compounds with short side chains on the quinolinium rings or having a phenanthroline group instead of the pyridine central ring, seem too bulky for the efficient intercalation into the VK2.

#### 2.2.3. Quinoline Derivatives with Olefin Linkers

Recently, two different NMR solution structures of the *c-MYC* promoter G4 2345 (WT and G23T mutant, with PDB reference 7KBX and 7KBW) complexed with a quinoline derivative bonded by an olefin linker to a phenyl group (named as PEQ) were solved [182]. In both these structures (Figure 11D and Table 5), the reported 2 ligands: 1 G4 complex evidenced a strong π–π interaction between the quinoline and phenyl moieties of the ligand and two G-tetrad guanines, in both 5′ and 3′ ends. Interestingly, the point mutation of G23 found at the 3′ end, previously shown as key for the selective binding to c-MYC G4s [191], was shown to highly affect the PEQ interactions with this G4 due to the structural chemical differences of the thymine in respect to the guanine. While in the WT c-MYC structure, the PEQ molecule interacts with the sugar edge of the G23, in the mutated T23, it stacks over the ring of G22. Additionally, the PEQ molecule in complex with the mutant c-MYC is rotated about 30° along the central G4 axis, partially covering G18. The observed differences in the mutant c-MYC place the carboxamide group on the quinoline ring in the G13/G18 groove. At the same time, in its WT version, this group is shifted towards the G9/G13 groove. Moreover, the hydroxyl and methoxy groups of the phenyl ring flank the G9 sugar in the mutant c-MYC. Notwithstanding, both groups are located in the G9/G22 groove in WT c-MYC G4, suggesting that both positions of the PEQ molecule can be used as promising sites for rational selective design towards different G4s.

### 2.3. Macrocycles

**Figure 12:**
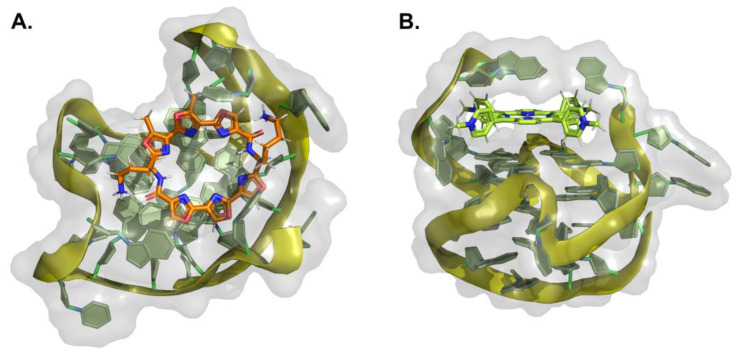
Structural representations of the complexes between different macrocycles and G4s. (**A**) top view of telomestatin derivative **3,3-L2H2-6OTD** interacting with the telomeric DNA G4 (PDB 2MB3). (**B**) side-view of a cationic porphyrin derivative interacting with a parallel DNA G4 (PDB 2A5R). All the ligands are represented as colored sticks, while the G4s are represented as cartoon and transparent surfaces.

NMR (Figure 12) and X-ray crystallography (Table 5) were also used to determine the 3D structures of the complexes established between G4′s and the two macrocyclic chemotypes: telomestatin and porphyrins.

#### 2.3.1. Telomestatin

Telomestatin (Figure 6) is a naturally occurring compound isolated from *Streptomyces anulatus* 3533-SV4 [255]. Due to its low toxicity and high inhibitory effect over telomerase function, this compound was initially seen as a promising anticancer drug lead. Additionally, telomestatin was also reported to selectivity interact and bind to G4s over duplex and single-stranded DNA [256]. Despite the problems associated with its synthesis, functional group substitution, and low solubility, one derivative containing two alkylamine side chains and six oxazole rings (**3,3-L2H2-6OTD** in Figure 15) was shown to overcome both previously mentioned problems and at the same time evidence a high affinity to the hTelo DNA G4 [257,258]. The complex of this telomestatin derivative with the parallel hTelo DNA G4 is currently available at the Protein Databank through the PDB code reference 2MB3 [259]. As shown in Figure 12A, this ligand interacts with the top G-quartet through π-stacking, while the two cationic flexible side chains face the negatively charged phosphate backbone of the G4. Under the established complex, it is important to mention the positions of the two methyl groups on the oxazoles, above a wide groove, away from the bases at the 5′ end. Considering the interaction topology of this telomestatin derivative and the G4, it became highly consensual that although planarity is essential in binding ligands to G4, the development of derivatives with higher flexibility and refined lengths of the cationic side chains should improve the affinity and selectivity towards specific G4 structures.

#### 2.3.2. Porphyrins

The porphyrin **TMPyP4** (Figure 6) is widely considered a potential photosensitizer in photodynamic therapy and an efficient G4 stabilizer for cancer therapeutics [31,260] due to its high-water solubility, high permeability through the cell membrane, and preferential accumulation in tumor cells. Currently, at the RCSB databank, several 3D structures of this class of compounds have been resolved in complex with different DNA and RNA-based G4 topological arrangements (Table 5). Independently of the type of G4 topology, this class of compounds stacks on the top of the G-quartet core through the typical π–π stabilization interactions with the guanines. As can be seen from the PDB structure with reference 2A5R (Figure 12B) [261], the interaction between these compounds and G4′s is further stabilized by the cationic aromatic rings hanging from the porphyrin core.

In addition, one can also find at the RCSB databank structures of methylmesoporphyrins complexed with parallel hTelo DNA G4. This type of porphyrin ligand is exceptionally selective for this topological arrangement of the G4. In the available structures at the protein databank (4G0F [262], 4FXM [262], and 6PNK [263]), it is possible to see that this sub-class of compounds binds with high affinity to the terminal G-quartet through π–π stacking. The out-of-plane *N*-methyl group of these ligands typically fits perfectly into the center of the parallel G4, where it aligns with the positive potassium ions. In contrast, the interaction of the *N*-methyl group with duplex DNA or antiparallel G4s would lead to steric clashes preventing them from binding to these structures, thus explaining its unique selectivity. Interestingly, the N-methylmesoporphyrins’ interaction with G4s does not rely on relatively nonspecific electrostatic interactions, which characterize most canonical G4 ligands, but rather through their hydrophobic nature. Even the carboxylate groups of the propionates seem to participate in a nonspecific stabilizing flexible hydrogen-bonding network with the backbone phosphates, granting an additional complex stabilization.

**Table 5:** Summary table with reference complex structures of G4s with modular aromatic ligands and macrocycles available at the RCSB Protein Databank.

PDB ID	Method	Ligand chemotype	Quadruplex	Refs.
**6KFJ**	NMR	tri-arylamine	Hybrid hTelo DNA g4	[246]
**6KFI**	NMR	tri-arylamine	Hybrid hTelo DNA G4	[246]
**2JT7**	NMR	pyrrole carboxamide	Parallel DNA G4	[248]
**2KVY**	NMR	pyrrole carboxamide	Antiparallel dimeric DNA G4	[249]
**5W77**	NMR	benzofuran (DC34)	*c-MYC* parallel DNA G4	[181]
**6SX3**	NMR	pyridine carboxamide (360A)	AGCGA- DNA quadruplex	[250]
**7KBX**	NMR	quinoline with olefin linker	*c-MYC* 2345 T23 mutant DNA G4	[182]
**7KBW**	NMR	quinoline with olefin linker	*c-MYC* 2345 parallel DNA G4	[182]
**2MB3**	NMR	Telomestatin (3,3-L2H2-6OTD)	Parallel hTelo DNA G4	[259]
**2A5R**	NMR	Porphyrin (TMPyP4)	*c-MYC* parallel DNA G4	[261]
**2HRI**	X-ray	Porphyrin	Parallel hTelo DNA G4	[264]
**4G0F**	X-ray	Mesoporphyrin	Parallel hTelo DNA G4	[262]
**4FXM**	X-ray	Mesoporphyrin	Parallel hTelo DNA G4	[262]
**6JJI**	X-ray	Mesoporphyrin	Two-quartet parallel RNA G4	[265]
**6JJH**	X-ray	Mesoporphyrin	Two-quartet parallel RNA G4	[265]
**6PNK**	X-ray	Mesoporphyrin	Parallel DNA G4 dimer	[263]
**6P45**	X-ray	Mesoporphyrin	Parallel DNA G4 dimer	[263]

## 3. Ligand-Induced Effects

The interaction of many small molecules with quadruplex structures, mostly with G4s, has been studied in the past decades [175,266,267], using different biophysical methods as recently reviewed by Cruz et al. [268] and also discussed in the previous sections. Moreover, many other in-vitro methods have also been used to study the effect of these molecules on the G4-protein interactions and how this translates into induced changes in genome regulation [269]. The understanding of how quadruplex-interactive small molecules bind and induce (or not) an effect on the quadruplex structure is of crucial importance for the development of the desired therapeutic agents, or better and more specific probes to study the function of quadruplexes in cells, or to be used in quadruplex-based nanodevices. In this section, we will summarize the state of the art on the reported ligand-induced effects on G4s and iMs using selected examples.

### 3.1. Ligand-Induced Quadruplex Stabilization or Destabilization

**Figure 13:**
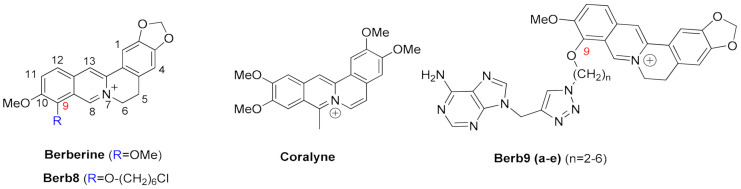
Structures of the G4 stabilizers Berberine, Berb8, Coralyne and Berb9 (a-e).

Most G4-interactive small molecules were reported as G4 stabilizers [175,267,269] and those with a fused polycyclic aromatic skeleton showed, in in-vitro assays, higher binding affinities and G4 stabilization efficacy. As seen in previous section, this type of G4 ligands most frequently binds to the external G-quartets through π–π interactions between the planar large aromatic core of the ligand and the guanines of G-quartet. Some of these ligands are natural alkaloids. Among them are **berberine**, a naturally occurring isoquinoline alkaloid which 3:1 complex with hTelo DNA G4 dimer has already been determined by X-ray crystallography (Figure 8C and Table 4). The structure of berberine (Figure 13) has undergone several chemical modifications in appropriate positions, namely at the 8, 9 and 13 positions of the isoquinoline core. Berberine was first reported to inhibit telomerase activity [270]. In addition, the analogue **coralyne** (Figure 13) stabilizes telomeric G4s and inhibit telomerase activity [271]. Regarding the thermal stabilization of a preformed intramolecular G4, a study revealed that these compounds are capable of stabilizing, instead of inducing, the intramolecular structure of the G4. The authors suggest that berberine derivatives are selective in inducing intermolecular G4 over intramolecular G4. In both cases, they found that **coralyne** is the best G4 stabilizer. In the TRAP assay (telomeric repetition amplification protocol), **coralyne** is also the most potent telomerase inhibitor with an IC_50_ value of ca. 70 µM [271].

To enhance the interaction of ligands with G4, 9-*O*-alkylamine or 9-*N*-alkylamine berberine derivatives were synthesized. The results showed that berberine and its 9-*O*-substituted derivatives could induce and stabilize the anti-parallel G4 of telomeric DNA d[G3(T2AG3)_3_] (telo21), in the presence or absence of metal cations. When compared to berberine, these 9-*O* derivatives, exhibit stronger binding affinity to G4 and among them, derivatives with a propylamine side chain were found to have higher affinities. In addition, authors conclude that berberine derivatives that are more effective stabilizers of the G4 structure are also better inhibitors of the telomerase activity [272]. The interaction of 9-*N*-substituted berberine derivatives with a DNA G4 present in the promoter region of *c-MYC* was also evaluated [273]. The results showed that these derivatives could selectively induce and stabilize the parallel intramolecular G4, resulting in the negative regulation of *c-MYC* transcription in the HL60 lymphoma cell line.

More recently, the same research group synthesized another 9-*O*-substituted berberine derivative, **Berb8** (Figure 13), with a chlorohexyl side chain at the 9-position [274]. Since the t-loop in telomere is stabilized by a protein complex of six subunits called shelterin, and three of them, TRF1, TRF2 and POT1, directly recognize the repetitions TTAGGG, the authors reported that this ligand induce the delocalization of TRF1 and POT1 from the telomere, accompanied by a rapid telomere uncapping, which may be the main mechanism of antitumor action of **Berb8**. On the other hand, using the BG4 antibody, which was used for quantitative visualization of DNA G4 in human cells, the authors revealed that **Berb8** could increase the amount of G4s in-vivo. In order to deduce a relationship between the chain length and binding properties, adenine–berberine derivatives **Berb9(a-e)** (Figure 13) with a variable length linker were synthesized [275]. The rationale of this research was based on the fact that adenine unit could establish additional interactions with the G4 loop region, through Watson-Crick bases pairing with the thymine residues. The association of the synthesized berberine–adenine conjugates with the representative quadruplex-forming oligonucleotides 22AG dA(G3TTA)_3_G3 and a2 d(ACAG4TGTG4)_2_ was investigated and led to the conclusion that there was the formation of 2:1 or 1:1 complexes (ligand:G4-DNA) with log K_b_ values of 10–11 (2:1) and 5–6 (1:1), values that are similar to those described for other berberine derivatives [276]. However, the authors found that there is no clear relationship between the binding affinity of the ligands and the length of the alkyl chain. Another conclusion of this work was that the triazole ring, used as a connection unit, causes steric impediment and restricts conformational flexibility in the vicinity of the adenine unit, thus making it difficult to bind to the thymine residues in the loops.

Besides the applicability of such compounds as therapeutic agents, berberines have also been looked as promising fluorescence probes. The parent **berberine**, shows a low emission quantum yield in aqueous solution which increases significantly upon binding to nucleic acids [277,278]. However, in most cases, this light-up effect is merely coincidental between emission and DNA-binding properties. Nevertheless, 9- and 12-dimethylaminophenyl-substituted berberine derivatives showed potential as pH dependent probes for optical detention of G4s [279]. Moreover, the derivatives **Berb9** upon binding to DNA G4 also exhibited pronounced fluorescent light-up effects, namely, with the alkyl chain *n* = 4 (with a2) and *n* = 5 (with 22AG) [276].

A review of the literature leads us to conclude that the interaction of **berberine** and derivatives with G4s has been studied in detail, but the interaction of these compounds with iMs has received less attention. As mentioned in Section 1, recent studies have suggested that G4s and iMs can each control specific biological activities in cells. Therefore, agents that interact with iMs will be important tools to investigate their functions in the cell’s genome as well as potential novel therapeutic agents [13,107,280,281]. Fluorescence spectroscopy and circular dichroism (CD) spectroscopy studies showed that **berberine**, as a fluorescence probe, can generate a turn-on response upon binding to an iM. In view of these results the authors proposed **berberine** as a novel light-up iM fluorescence ligand [278]. However, later works questioned this proposal of **berberine** as a selective probe for iMs. The Randazzo group investigated the interaction of some known G4 ligands, including **berberine**, with the iM structure formed by the sequence 5′-CCCT (AACCCT)_3_-3′, corresponding to the end of the human telomere [282].

The combination of different spectroscopic techniques allowed the authors to conclude that **berberine** is able to interact with the iM forming DNA but do not have relevant effects on the thermal stability of iM. In addition, studies carried out with the labelled C-rich strand EGFR-272_C at pH 5.5 and at pH 7.4, to verify the stabilizing properties in the folded iM and the ability to induce the iM structure at physiological pH, respectively, revealed a lower ability of **berberine** to recognize or induce the structure of iM [283]. In order to provide a further insight into the interaction mode of **berberine** with iMs, another study characterized the interaction of this ligand with DNA iM forming sequences found in the human telomeres [281]. NMR spectra of oligonucleotides showed sharp peaks at 15–16 ppm region, characteristic of iM formation, but no significant chemical shift variations were observed after the addition of increasing amounts of **berberine**. In accordance with all spectroscopic data, the authors conclude that the interaction of **berberine** with iMs is low, primarily electrostatic, and occurs with bases not involved in C.C^+^ base pairs. Furthermore, **berberine** is not selective for iM structures because it also binds to folded and unfolded cytosine-rich sequences. In conclusion, as summarized in Table 6, the authors suggest that the main event responsible for the biological activity of **berberine** is probably due to its high affinity to G4 and not to the interaction with iM structures.

**Figure 14:**
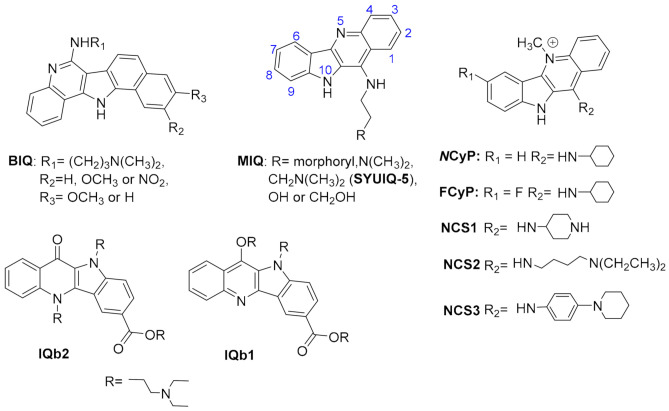
Structures of benzoindoloquinolines and indolo[3,2-*b*]quinoline derivatives.

Another class of G4-interactive natural alkaloid is based on the indoloquinoline scaffold (Figure 14) [284,285,286]. One of the first reports on these compounds disclosed that benzoindoloquinolines derivatives, **BIQ** (Figure 14), can stabilize and induce the formation of an intramolecular G4, and the increase in melting temperature of the G4 was associated with telomerase inhibition in-vitro [287].

Other studies exploited the indolo[3,2-*b*]quinoline (or quindoline) tetracyclic aromatic core (Figure 14). Derivatives with one or two aminoalkyl substituents at C2, C7, C11 and N10 were shown to be potent and selective stabilizers of hTelo and oncogene promoter DNA G4s, as well as telomerase activity inhibitors and c-MYC expression downregulators [288,289,290,291,292,293]. In the case of monosubstituted indolo[3,2-*b*]quinoline derivatives **MIQ** (a-e) (Figure 14), in which an electron donor amine group was introduced in position C-11, it is believed that this change significantly increases the basicity of the nitrogen in the quinoline ring, which favours the electrostatic attraction between the ligands and the negative electrostatic center of the G4 [290]. In fact, the complex of **SYUIQ-5** with the c-MYC promoter G4 determined by NMR (Figure 8B) corroborates this suggestion.

Another approach was the *N*-methylation at the 5-position to yield cryptolepine analogues, with a stable positive charge on the aromatic core to improve the binding ability to G4 [294]. Results from CD spectroscopy, FRET melting assay and PCR stop assay led the authors to conclude that compounds **NCyP** and **FCyP** could induce the formation of antiparallel hTelo DNA G4 in the presence of K^+^. NMR studies and molecular modelling revealed that the binding mode was external stacking on the G-quartet, and the positively charged at the 5-N position of the quindoline core could contribute to the stabilization capacity [294]. Another study reported the in-vitro and in-vivo anticancer profiles of selected C-11 diamino alkyl cryptolepine derivatives, **NCS** (Figure 14) [295]. FRET melting assay using a G4 forming 21nt hTelo DNA sequence revealed that the C-11 derivatives are effective G4 DNA ligands, with ΔTm values of 21 °C and 20 °C for **NSC2** and **NSC1**, respectively. However, for compound **NSC3**, in which an aromatic amine side chain was introduced at position C-11 of the cryptolepine structure, the value of ΔTm decreases dramatically (ΔTm = 4 °C). Although compounds **NSC1** and **NSC2** are good G4 stabilizers, they also showed low selectivity when compared with the duplex DNA binding (Q/D < 2). To improve the selectivity of these ligands, 20 indolo[3,2-*b*]quinolines mono-, di-, and trisubstituted with basic side chains were designed, synthesised, and evaluated for thermal stabilization of three different DNA G4s and a hairpin duplex DNA [296]. Compounds **IQb2** and **IQb1** (Figure 14) revealed 10-fold selectivity for G4 over duplex DNA, and 100-fold selectivity for the HCT116 cancer cell line (IC_50_ and IC_90_ < 10 µM) over primary rat hepatocytes. Moreover, the trisubstituted ligands **IQb2** and **IQb1**, clearly improved stabilization of DNA G4s comparing with disubstituted derivatives and the following inter-G4 thermal stability trend effect was observed: Hsp90A > KRas21R~F21T > c-Kit2 > T-loop. In addition, these compounds downregulated the expression of oncogene *KRAS* in colon cancer cells by the inhibition of both transcription and translation, as mentioned in Section 1.

Additionally, the regioisomeric indolo[3,2-*c*]quinolines (**IQc**) with one or two alkylamine side chains and a N5-methyl group have been investigated for their binding mode and efficiency to stabilize DNA G4s [297,298]. The FRET melting assay revealed that **IQc3d** with two alkylamine side chains in positions 2 and 9 (see structure in Figure 22) is a better DNA G4 stabilizer than its mono- (**IQc2d**) and 2,8-disubstituted analogues. Fluorescence assays performed to understand the binding behaviour of this ligand to hybrid hTelo and parallel DNA G4s, revealed the binding of two **IQc3d** molecules per G4 unit, in two non-independent but equivalent binding sites. Moreover, competition assays also showed that G4 ligand **IQc3d** shows a 50-fold selectivity for DNA G4s when compared with duplex DNA and a preference for the parallel G4 present in the promoter region of *KRAS* when compared to the hybrid hTelo G4 [297]. This study also showed that **IQc2d** and **IQc3d** are good stabilizers of DNA G4s, but only moderate stabilizers of RNA G4s UTR1 and UTR2.

Many acridines with potential therapeutic application (Table 1, Table 2 and Table 3) or with photochemical properties have been developed [299]. While disubstituted acridines can bind to both G4 and duplex DNA, the tri-substituted acridine **BRACO-19** (Figure 4) can effectively bind and stabilize G4s with negligible binding affinity for duplex DNAs [299,300,301,302]. Like acridines, berberines and indoloquinolines, also phenanthrolines, naphthalene diimides, quinolones and other G4 ligands in Figure 4, are potent and selective G4 stabilizers when compared to dsDNA. Interestingly, some of these G4 stabilizers were also reported to interact and destabilize DNA iMs (Table 6). This is the case of **BRACO-19**, **PhenDC3** (Figure 6) and **nitidine** (Figure 4) [108,282].

**Figure 15:**
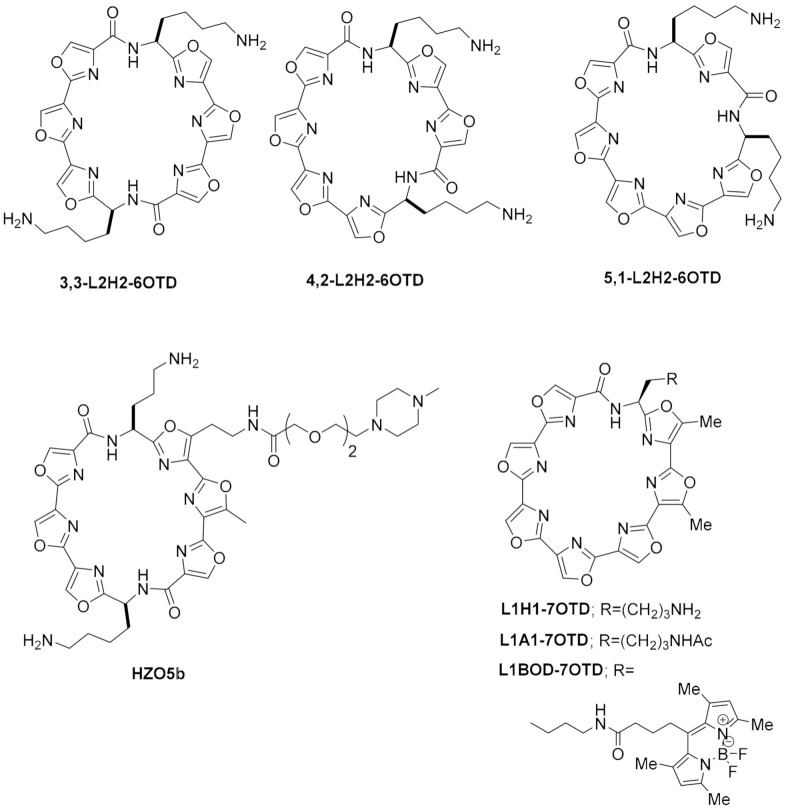
Structures of macrocyclic hexa- and heptaoxazoles.

Macrocyclic structures that have been modelled from the natural product **telomestatin** or are porphyrin-based (e.g., **TMPyP4**) compounds (Figure 6), have given rise to some of the most potent and selective G4 ligands currently known. **Telomestatin**, a macrocyclic compound containing five oxazoles, two methyloxazoles, and a thiazoline ring, has been reported as a highly potent and selective telomerase inhibitor, giving an IC_50_ value in the TRAP assay of 5 nM [255,303]. Moreover, the FRET melting assay revealed that telomestatin is a good stabilizer of DNA G4 (ΔT = 24 °C) but shows low affinity for double-stranded DNA [304]. Since most cancer cells depend on telomerase for their survival, this macrocycle would be an attractive target in anticancer drug design. However, its high hydrophobicity and low solubility, combined with the lack of efficient synthetic routes, prevented this compound from reaching the market. To overcome these problems, the synthesis of oxazole-based telomestatin derivatives (**OTD**s) were developed. Among them, macrocyclic hexaoxazole compounds bearing symmetrical (**3,3-L2H2-6OTD**) and unsymmetrical side chains (**4,2-L2H2-6OTD** and **5,1-L2H2-6OTD**) were synthesized and evaluated as G4 ligands (Figure 15) [257,305]. Compound **3,3-L2H2-6OTD** strongly stabilize the antiparallel telo24 DNA G4 with a ΔTm value of 18.8 °C, while compounds **4,2-L2H2-6OTD** and **5,1-L2H2-6OTD** show very low stabilizing effect, with ΔTm values of 7.9 and 4.1 °C, respectively. These results were better understood through molecular mechanics calculation, in which, it is shown that the planarity of the asymmetric macrocyclic moieties in **4,2-L2H2-6OTD** and **5,1-L2H2-6OTD** was decreased in comparison with the symmetrical structure of the ligand **3,3-L2H2-6OTD**. Moreover, most of these compounds selectively stabilize G4 structures since no significant variation in melting temperature was obtained for dsDNA in the presence of these macrocycles. The complex of **L2H2-6OTD** with hTelo DNA G4 was already determined by NMR (PDB ID 2MB3; Figure 12B). In addition, a series of macrocyclic hexaoxazoles bearing three side chains were synthesized (Figure 15) [258]. Results from FRET melting assay revealed that compound **HZO5b** preferentially stabilizes telomeric G4 over the G4-forming DNA sequences of *c-KIT* and *KRAS* promoters. Moreover, molecular docking studies between parallel telomeric DNA G4 and the ligand **HZO5b**, showed that the hexaoxazole moiety stacks with the G-quartet, and the newly introduced piperazinyl alkyl side chain is suggested to interact with the groove of the G4, probably through interactions established between the two piperazine nitrogens and the DNA phosphate backbone.

Another strategy for improvement consisted on the dimerization of the 6OTD scaffold, using a bisamide linker between the two 6OTD moieties [306]. The dimer **HZOD10** showed potent G4 stabilizing activity with the two interconnected macrocycles interacting as terminal caps of the outer quartets of the same G4 structure. The dimerization of the 6OTD structure, in particular for dimer **HZOD10**, increases the selectivity for interaction with telomeric DNA at least 10 times.

A series of macrocyclic heptaoxazoles, were also synthesized. The heptaoxazole based macrocycles were synthesized in order to increase the structural planarity of the ligands (Figure 15) [307]. Both compounds, **L1H1-7OTD** and **L1A1-7OTD,** interacted with telo24 G4 and induced formation of a typical antiparallel structure. These heptaoxazole macrocyclic structures showed high selectivity for G4-forming sequences, and the selectivity of **L1A1-7OTD** for telo24 over its mutant non-G4-forming oligonucleotide was more than 200-fold. In addition, the authors placed a fluorophore on the side chain of the 7OTD, giving rise to a fluorescent G4 ligand, **L1BOD-7OTD**, with which it was possible to visualize the G4 structures in the cells. Since the planar structure of iM is smaller than that of G4, the Nagasawa group synthesized penta- and tetra-oxazoles (L2H2-5OTD and **L2H2-4OTD**) bearing amine functional groups in the side chains [107]. These compounds revealed a reduced G4 stabilizing ability but on the other hand, the tetra-oxazole compound interacted with iM sequences. Results from electrospray mass spectroscopy confirmed that two molecules of the tetra-oxazole compound are involved in this interaction, and these molecules bind cooperatively to the iM structure.

**Figure 16:**
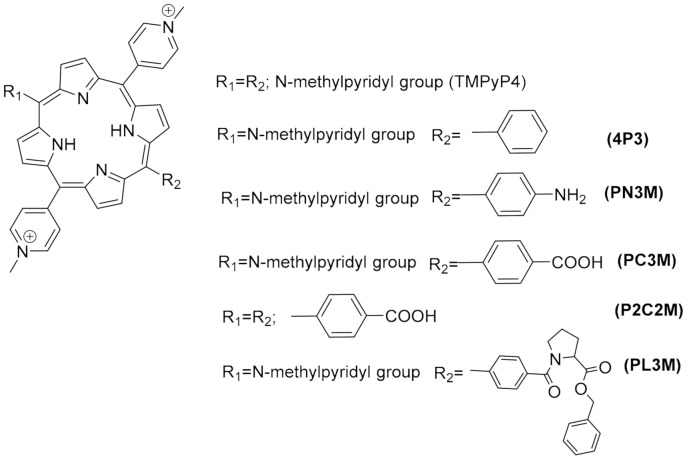
Structures of TMPyP4 derivatives.

Another class of macrocyclic G4-interacting compounds are porphyrins and related pyrrole-based structures. One of the most intensively studied G4 ligands is the cationic 5,10,15,20-tetra(N-methyl-4-pyridyl)porphyrin, **TMPyP4** (Figure 6). Hurley’s group showed for the first time that **TMPyP4** binds and stabilizes the hTelo and c-MYC promoter DNA G4s, as also inhibits telomerase activity and down-regulates *c-MYC* expression [31,308]. Several studies have been performed to gain insights on porphyrin **TMPyP4** and its positional isomers **TMPyP3** and T**MPyP2** (Figure 6) regarding G4 binding, inhibition of telomerase activity and anticancer effects [308,309,310,311,312]. In addition, **TMPyP4** was found to promote the formation of the iM structure [313]. However, the binding mode and stoichiometry of **TMPyP4** to G4 structures, as reviewed elsewhere, is not consensual [314]. Nevertheless, the 1:1 complex of this macrocycle with the parallel c-MYC DNA G4 has already been resolved by NMR (Figure 12B). However, the porphyrin **TMPyP4**, binds strongly to a wide range of G4 nucleic acids, as well to duplex and other nucleic acid forms, thus revealing a low selectivity [315]. To improve the selectivity over G4, five derivatives of TMPyP4 were synthesized (Figure 16), where one *N*-methylpyridyl group was replaced with a phenyl (**4P3**), a 4-aminophenyl (**PN3M**), a 4-phenylamidoproline (**PL3M**), a 4-carboxyphenyl (**PC3M**) and two *N*-methylpyridyl groups replaced by two 4-carboxyphenyl groups (**P2C2M**) [316].

All porphyrin derivatives, apart from **P2C2M**, stabilize human telomeric DNA (Tel22) in FRET assays (at 5 eq) by ~20 °C. Interestingly, the binding constants determined using UV-vis titrations, correlate well with the charge: triple cationic **4P3**, **PN3M** and **PL3M** display Ka of 5–9 µM^−1^, double cationic **PC3M** displays Ka of 1 µM^−1^, and neutral **P2C2M** displays a weak-to-no binding affinity. Through the data obtained by UV-vis, the authors suggested that the binding is driven by electrostatic interactions and base stacking (or end-staking). Interestingly, this study led to the conclusion that by decreasing the charge on **TMPyP4** to 3^+^, the desired balance between the stabilization capacity, affinity and high selectivity required for an excellent G4 ligand can be achieved [316,317]. Although **TMPyP4** is known mainly as a DNA G4 stabilizer, there are also studies reporting this ligand as a G4-disrupting small molecule. Weisman-Shomer et al., disclosed that in vitro this ligand effectively causes a destabilization of the G’2 bimolecular quadruplex structure related to Fragile X FMR1 gene, containing d(CGG) repeats. Results showed that **TMPyP4** decreased by ~15 °C (at 0.3 µM) the thermal stability of the d(CGG) trinucleotide repeat [318]. Another more recent study also reported the destabilization by **TMPyP4** and **TMPyP3** of a parallel DNA G4 formed by a 33-mer sequence present in the human multidrug protein 1 (MRP1) transporter gene [319].

It was also demonstrated by CD spectroscopy that **TMPyP4** unfolds an unusually stable RNA G4, in a concentration-dependent manner [320]. Moreover, **TMPyP4** binds and distorts RNA G4 formed by the C9orf72 (GGGGCC)n repeat, whose expansion is related to Amyotrophic lateral sclerosis and frontotemporal dementia. Its interaction also disrupts the binding between the G4 and ASF/SF2 and hnRNPA1 proteins [321]. Recently, Singh et al. also observed a destabilizing effect of porphyrins **TMPyP4** and **TMPyP3**, on the parallel HM23G4 [322]. HM23G4 formation takes place in a 23-nt G-rich promoter sequence of myosin gene, associated with various cardiac related diseases. Among both ligands, **TMPyP4** displays the best destabilizing and binding properties towards HM23G4. The addition of porphyrins **TMPyP3** and **TMPyP4** (4 equivalents) destabilizes the quadruplex structure by 17 °C and 34 °C, respectively. The authors, based on the data acquired by UV-vis, CD and fluorescence, suggested that being planar, porphyrins interact via end stacking and intercalate between adjacent G-tetrads disrupting the π–π interactions and thus destabilizing HM23G-quadruplex.

**Figure 17:**
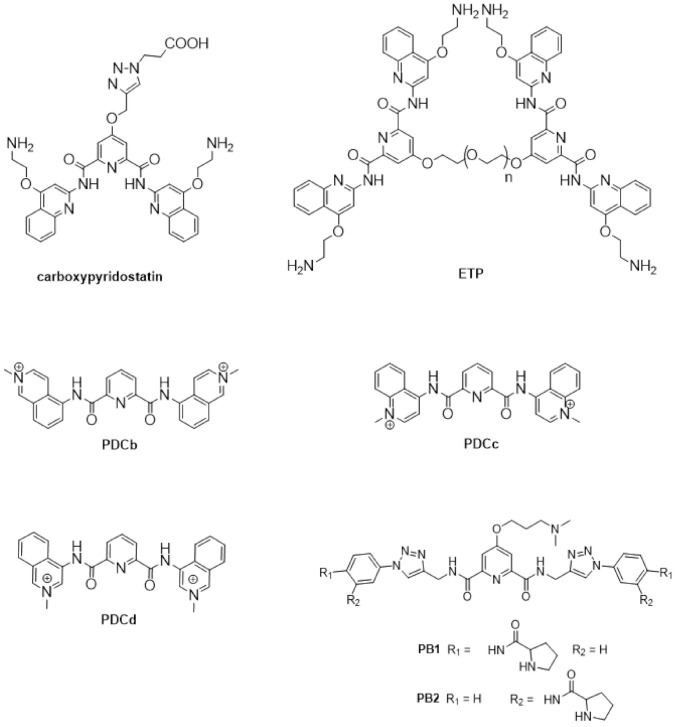
Structures of pyridine-2,6-dicarboxamide derivatives.

Ligands based on bis-quinoline dicarboxamides scaffold, like **pyridostatin** and **PDC-360A** (Figure 6), are known to be potent G4 stabilizers [178]. An extensive review on the efficiency of G4 bind and selectivity of pyridostatin derivatives has already been published [64]. Interestingly, the derivative carboxypyridostatin (**carboxyPDS**) (Figure 17), exhibits high molecular specificity for RNA over DNA G4s [59]. In addition, **carboxyPDS** increases the number of G4s only in the cytoplasm, which suggests that in cells this ligand selectively traps and stabilizes cytoplasmic RNA G4s and not nuclear DNA G4s. Recently, three new polyether-tethered pyridostatin dimers **ETP**(**a**-**c**) (Figure 17) were synthesized and their interaction with hTelo G4 dimers (G2T1) has been evaluated [323]. The high binding constants (K_a_ > 10^7^μM^−1^) of dimers **ETPb** (*n* = 2) and **ETPc** (*n* = 3) towards G2T1 reveal that they are two excellent binders. The dimer **ETPb** (*n* = 2) had higher binding affinity and selectivity towards antiparallel telomeric multimeric G4 than towards monomeric quadruplexes.

In order to study the influence of the position of carboxamide linker in the quinoline/isoquinoline rings on the stabilization of DNA G4s, new **PDC-360A** isomers (**PDCb-d**) were studied (Figure 17) [183]. Among these, compounds having a relative 1,3-position between the charged methylquinolinium/isoquinolinium nitrogen and the amide linker (**PDC360A** and **PDCd**) showed to be the best DNA G4 stabilizers. Molecular modelling studies with a parallel DNA G4 suggest that **PDC-360A** (Figure 6) and **PDCd** can adopt an almost planar conformation and stack through π–π interactions with the top G-quartet, whereas the worst G4 stabilizer, **PDCb**, interacts preferentially with a groove/loop of the G4, due to its twisted configuration.

Another interesting study with the pyridine 2,6-dicarboxamide derivatives **PB1** and **PB2** (Figure 17) shows that small chemical changes may have a big impact on discrimination between quadruplexes [110]. The *para*-regioisomer **PB1** preferentially induces the formation and stabilizes the iM quadruplex present in promoter of *BCL-2*, whereas the *meta* regioisomer **PB2** has a preference to induce and stabilize the complementary G4 structure. Moreover, they induce different effects in cells: **PB1** upregulates and **PB2** downregulates the expression of BCL-2.

**Figure 18:**
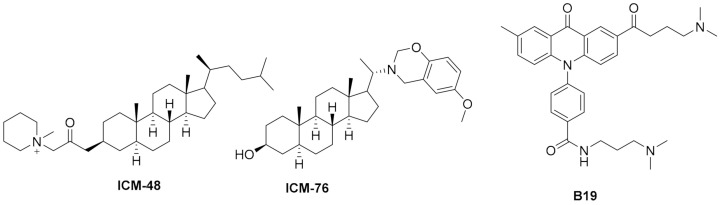
iM-interactive stroid derivatives ICM-48 and ICM-76, and acridone B19.

In a quest for iM-interactive small molecules, Hurley and co-workers screened a NCI library of 1990 compounds against *BCL-2* iM to discover 9 iM-destabilizing small molecules and 4 iM-stabilizing molecule [280]. Among these hit compounds are the steroids **ICM-48** and **ICM-76** (Figure 18), which were confirmed using other techniques, including NMR, to be iM-interactive compounds inducing different effects. **ICM-48** binds and stabilizes preferentially *BCL-2* iM, when compared to *VEGF*-iM and *c-MYC*-iM, whereas **ICM-76** also binds preferentially to *BCL-2* iM but destabilizes the quadruplex, shifting the equilibrium to the hairpin form. Moreover, the compounds also induced different effects in cells. The iM-stabilizer **ICM-48** induced an upregulation of the *BCL2* gene, whereas iM-destabilizer **ICM-76** decreased the expression of this gene. Interestingly, a secondary screening of an additional 14 steroidal compounds allowed the identification of eight more iM destabilizing compounds, but no other stabilizing compound was found. More recently, another group discovered the iM-interactive acridone **B19** (Figure 18), which binds and stabilizes the iM of the *c-MYC* promoter without significant binding to the complementary G4 or dsDNA. However, in this case, the *c-MYC* transcription and expression were downregulated in cancer cells treated with the iM stabilizer **B19** [109].

**Figure 19:**
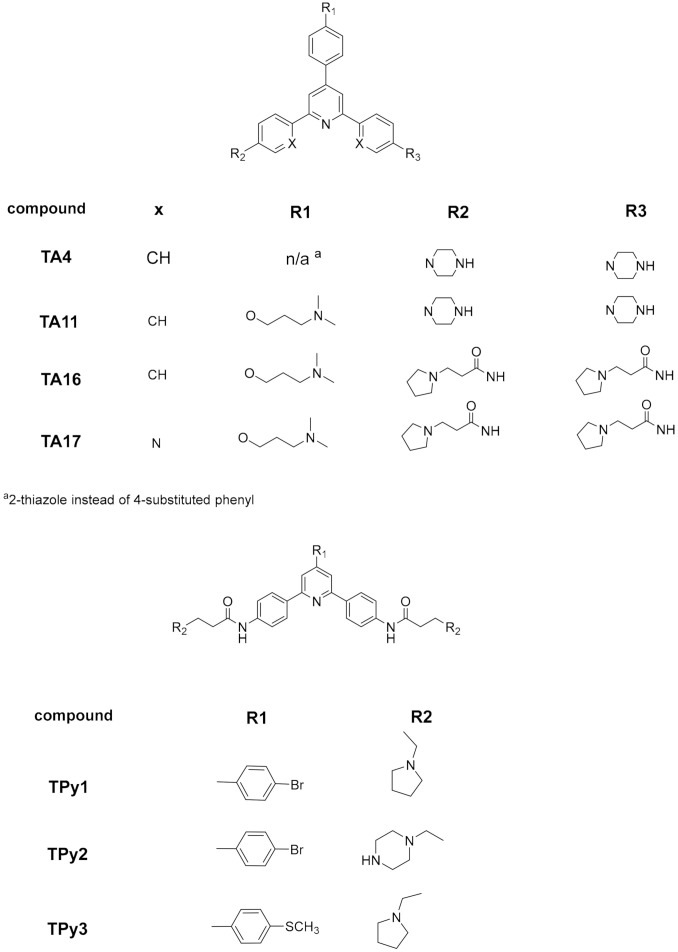
Structures of triarylpyridines and terpyridines.

Triarylpyridines and terpyridines (X = N) (Figure 19) are compounds that provide structural flexibility in their framework to target the loops and grooves of G4s, being an attractive template for ligand design. The triarylpyridines were reported as potent G4 ligands capable to discriminate between DNA G4 and duplex DNA [324]. Moreover, most of the compounds showed also a binding preference for the hTelo G4 when compared to the *c-KIT* promoter G4. For example, ligand **TA11** showed good binding affinities to both hTelo and c-KIT2 DNA G4s (Kd = 320 nM and 640 nM, respectively) and stabilization (ΔTm = 21 °C and 17 °C, respectively), with a preference for hTelo G4, like most of the triaryl and terpyridines studied. An exception to this trend is **TA4**, which showed a twofold preference of binding to c-KIT G4 (Kd = 180 nM). Moreover, not all good G4 binders are also good G4 stabilizers, and *vice versa*. An example is the **TA17** which shows a large ∆Tm for c-KIT G4 (23 °C) but a modest Kd (11 µM) for the same G4.

In addition, terpyridines (X = N) showed higher G4 stabilization capacity but worst binding affinities when compared to their benzene (X = CH) counterparts (e.g., **TA17** vs **TA16**). Interestingly, the same research group later discovered that compound **TA17** at higher concentrations (10–100 µM) is capable of disrupting the c-KIT2 G4 [325]. In addition, in-vitro experiments with a cell line that expresses c-KIT (HGC-27 cells) showed that the same ligand increased the expression of this oncogene.

Other ligands containing the 2,4,6- triarylpyridine module have also been studied [326]. Compounds **TPy1** and **TPy3**, which contain the C4-pyrrolidine side chain, and compound **TPy2**, which contains the C4 piperazine side chain, exhibited the highest stabilizations for all evaluated G4-DNA sequences (Figure 19). These studies suggest that the nature of the 4-aryl substituents along with side chains length governs the DNA G4 stabilization ability of the compounds. Therefore, for high stabilization, a C4 chain length is preferred over a C3 chain length, as well as a p-bromophenyl or p-thiomethylphenyl 4-aryl substituent on the central pyridine ring.

**Figure 20:**
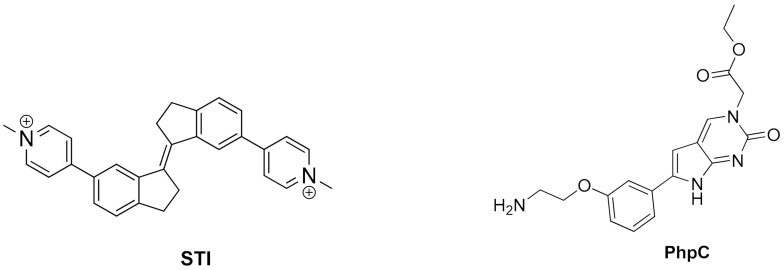
Structures of G4 ligands with capacity to disrupt the quadruplex structure.

The stiff-stilbene derivative **STI** (Figure 20) is a novel G4 binding agent that displays high affinity for DNA G4 with significant discrimination against duplex DNA [327,328]. In addition, this compound regulates the folding/unfolding of the telomeric G4. In other words, while the ligand stabilizes the hybrid form of telo23 G4 in K^+^ solution, induces the unfolding of the hybrid form of the same sequence in the presence of Na^+^ [327]. This effect can be reversed on-demand by irradiation with 400 nm light through deactivation of the ligand by photo-oxidation. Moreover, molecular dynamics and NMR studies suggested that **STI** binds to G4 grooves and intercalates between G-quartets, which may lead to disruption of the G-quartets H-bond network [327].

**Table 6:** Effects of selected G4 ligands on G4s and iMs, and their potential applications.

Ligand (Chemotype)	Effect on G4	Effect on iM	Potential Application	Refs.
**Berberine**	Stabilization (DNA G4)	Low and unspecific interaction	Anticancer drug	[270,278,281,282,283]
**Berb8** (berberine)	Stabilization (DNA G4)	-	Anticancer drug	[274]
**SYUIQ-5** (indolo[3,2-*b*]quinoline)	Stabilization (DNA G4)	-	Anticancer drug	[290]
**IQb2** (indolo[3,2-*b*]quinoline)	Stabilization (DNA G4)	-	Anticancer drug	[94]
**IQc3d** (indolo[3,2-c]quinoline)	Stabilization (DNA and RNA G4)	-	Anticancer drug	[297]
**BRACO-19** (acridine)	Stabilization (DNA G4)	Destabilization	Anticancer drug	[282,299,301]
**PhenDC3** (Phenanthroline)	Stabilization (DNA G4)	Destabilization	Anticancer drug G4 probe	[282]
**Nitidine** (Benzophenanthridine)	Stabilization (DNA G4)	Destabilization	Anticancer drug	[108]
**3,3-L2H2-6OTD** (oxazole-based)	Stabilization (DNA G4)	-	Anticancer drug	[305]
**L1BOD-7OTD** (oxazole-based)	Stabilization (DNA G4)	-	Fluorescent probe	[307]
**L2H2-4OTD** (oxazole-based)	Low stabilization (DNA G4)	Stabilization	-	[107]
**TMPyP4** (porphyrine)	Stabilization/destabilization (DNA G4) Destabilization (RNA G4)	Promotion and stabilization	Low potential application due to its low selectivity to quadruplexes	[308,313,318,319,320,321,322]
**pyridostatin**	Stabilization (DNA G4)	No effect	G4 probe	[282]
**CarboxyPDS** (pyridostatin)	Stabilization (RNA G4)	-	Anticancer drug	[59]
**TA17** (Terpyridine)	Stabilization at 1 µM (DNA G4) Destabilization at >10 µM (c-KIT2 G4)	-	Anticancer drug	[324,325]
**PB1** (pyridine dicarboxamide)	Low stabilization	Induction and stabilization	Therapeutic agent	[110]
**PB2** (pyridine dicarboxamide)	Induction and stabilization (DNA G4)	Low stabilization	Anticancer drug	[110]
**ICM-48** (steroid)	-	Stabilization	Therapeutic agent	[280]
**ICM-76** (steroid)	-	Destabilization	Anticancer drug	[280]
**B19** (acridone)	No interaction	Stabilization	Anticancer drug	[109]
**STI** (stilbene)	Regulates the folding/unfolding of the telomeric G4 in a photoresponsive manner	-	In nanodevices	[327]
**PhpC** (pyrrolopyrimidine)	Destabilization	-	Therapeutic agent	[329]

Very recently, the ability of a set of chemically diverse G4 ligands to disrupt G4 structures was investigated using several biophysical and biochemical methods [329]. The authors concluded that the effect of G4 ligands on the G4 structure is strongly dependent on the technique and concentration of the ligand, in agreement with a previous study [330]. Moreover, the study revealed a new G4 disrupting small molecule, the G-clamp analogue **PhpC** (Figure 20), whose ability to efficiently disrupt G4 structures facilitated G4-helicase activity [329].

### 3.2. Ligand-Induced G4 Topology Switches

As depicted in Figure 2, intramolecular G4s can form a variety of 3D structures depending upon their length and base sequences, as well as on molecular environment. Recently it became clear that the topologies of G4s have an important influence on G4-related biological function [331]. These functions are thought to depend on proteins that recognize the G4 structure, and since different topological forms of G4s have different binding affinities to their target proteins, the topology of G4s is considered to be biologically significant [331,332]. Therefore, G4 ligands that can control the topology of G4s should be extremely useful as chemical tools to elucidate the roles of G4 structures, and to further expand the possibilities of these compounds as therapeutics.

**Figure 21:**
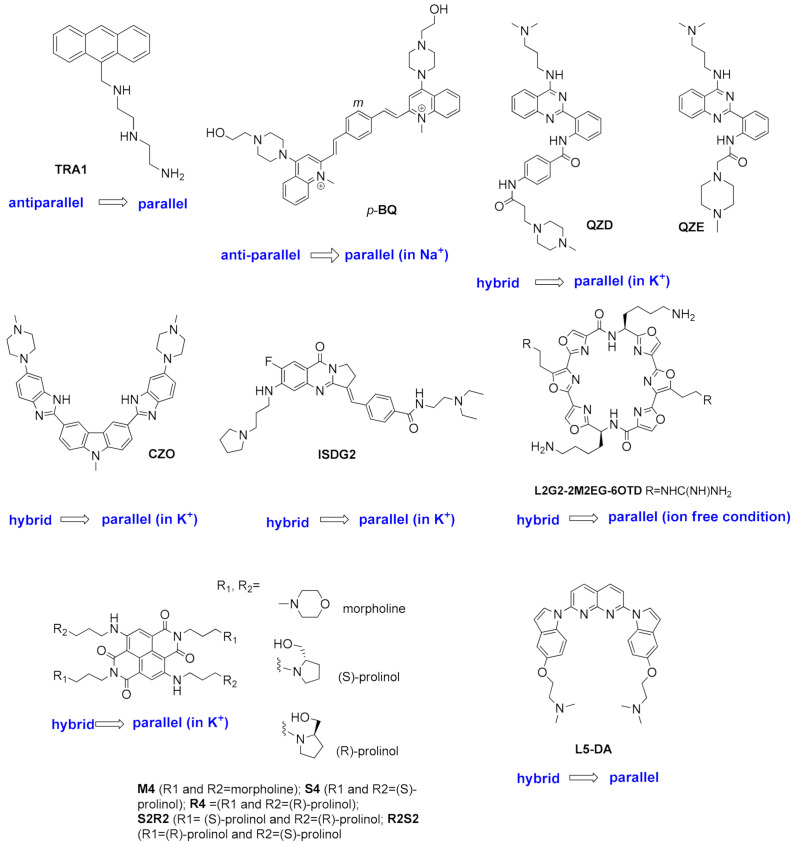
G4 ligands able to induce a topological switch to parallel.

**Figure 22:**
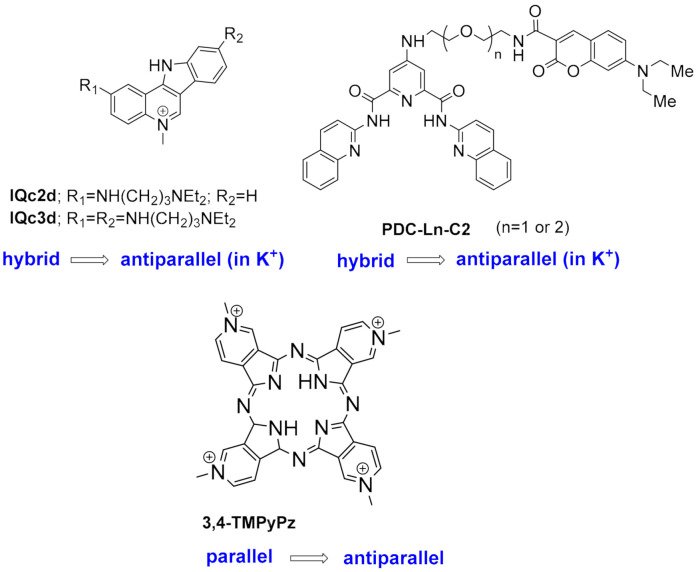
G4 ligands able to induce a topological switch to antiparallel.

Several research groups have focus specifically on small molecule ligands as G4 switching triggers. One of the first examples is the ligand **TRA1**, a triamino-anthracene derivative (Figure 21), which induces a conformational switch of the telo24 DNA G4 from an anti-parallel structure in Na^+^ buffer to a parallel form in about two hours. On the other hand, the porphyrazine derivative, **3,4-TMPyPz** (Figure 22) can induce and stabilize the anti-parallel DNA G4. Interestingly, the sequential addition of the two ligands (**TRA1** and **3,4-TMPyPz**) makes the G4 to switch between parallel and antiparallel, but the conformational switch was only reverse once in this manner [333].

Huang’s group synthesized 1,3- and 1,4-disubstituted benzene bisquinolinium derivatives (**BQ**), a *meta* and a *para* isomer of BQ (Figure 21), respectively. Both isomers could induce the telomere G4 DNA to form a parallel structure in co-annealed condition in presence of K^+^. However, only the *para* isomer could convert anti-parallel G4 to its parallel form in presence of Na^+^ [334].

In a previous report, the same group disclosed an interesting structure-activity relationship of a series of quinazoline derivatives. They demonstrated that ligands of type **QZD** (Figure 21) were able to induce a switch from hybrid type to parallel type G4, in potassium medium, while ligands of type **QZE** (lacking one benzene ring) were not able to exert such effect [335]. Another example is the carbazole derivative **CZO** (Figure 21), a new light up fluorescent probe, composed of benzimidazole and carbazole, that induces telo22 to adopt a parallel fold after being heat-denaturated and co-annealed with **CZO** in potassium buffer, rather than the usual hybrid-type G4 formed in these conditions [336]. The good affinity and selectivity for G4s of this ligand is attributed to the presence of the side arms of piperazine which are flexible and can form hydrogen bonds and electrostatic interactions with the phosphate backbone in the grooves and loops. This compound is a light up fluorescence probe in the presence of parallel DNA G4s but exhibits weak fluorescence with antiparallel DNA G4s. In addition, some isaindigotone derivatives, such as **ISDG2** (Figure 21) were unexpectedly found to induce the conformational switch of the hTelo DNA G4 from a hybrid topology to a parallel one in K^+^ solution. Encouraged by these results, they developed a computational study in order to generate a pharmacophore model that predicts the ability of these compounds to induce conformational changes of DNA G4s. A virtual screening of a database was performed using this pharmacophore model and new derivatives of benzopyranopyrimidine were identified as putative strong inducers of the telomeric conformation switch from hybrid to parallel structure [337].

Nagasawa and co-workers, inspired by the natural G4 ligand **telomestatin**, synthesized a series of macrocyclic hexaoxazoles as G4 ligands. In previous studies, they reported that the ligand **3,3-L2H2-6OTD** (Figure 15) bearing two n-butyl amino side chains, shows potent G4-stabilizing activity and induces the anti-parallel topology of the telomeric DNA sequence telo24 under ion-free conditions (Table 7) [305]. More recently, the same research group designed the ligand **L2G2-2M2EG-6OTD**, (Figure 21) with four side chains to target the four grooves of parallel DNA G4 [332]. The CD data allowed the authors to demonstrate that this macrocycle induces the formation of the parallel topology of telomeric and *BCL-2* DNA G4s, as well as of a thrombin G4 aptamer, which preferably forms hybrid and anti-parallel topologies. In addition, the authors demonstrated the ligand-induced switching process of telo24 DNA G4 from the anti-parallel or hybrid topologies to the parallel topology under ion-free conditions. Interestingly, the opposite direction of conversion, that is from parallel to antiparallel topology, is possible upon titration with **3,3-L2H2-6OTD**.

Based on some previously reported studies in which naphthalene diimide ligands (**NDI**) were shown to stabilize hTelo DNA G4 and inhibit telomerase activity as well as tumour cell proliferation, tetra-substituted NDI ligands were studied [338]. Model sequences Tel22 and Tel23 were selected, which are sequences of hTelo DNA that adopt hybrid topology when folding in K^+^ solution. The study on the interactions of G4 with **NDI** ligands revealed that the **NDI**s **M4**, **S4**, **R4**, **S2R2** and **R2S2** (Figure 21) can induce the conformational conversion of G4s from the initial hybrid topology to the parallel topology. The mechanism of **NDI**-induced conversion process was investigated at the cellular physiological temperature (37 °C), from low to high K^+^ concentration, and in a buffer or under mimetic cellular crowding conditions created by Ficoll 70. Three protocols were used for the preparation of DNA samples and deep insights into the topological conversion were obtained with NMR techniques. Using the data obtained with different spectroscopic methods, the authors proposed a mechanism for the switch of Tel23 DNA G4 in K^+^ solution, in which the topological conversion goes from the gradual unfolding of hybrid G4, through intermediate states, to the final parallel G4 [338]. In a recent report, the combination of naphthyridine and indole scaffold led to the development of a ligand **L5-DA** (Figure 21) that induced the conformational conversion from hybrid to parallel topologies. Following the addition of **L5-DA**, the melting temperature of Tel26 increased by more than 37 °C, indicating a significant improvement in the thermal stability of the resulting parallel Tel26 G4 [339].

Some research groups have reported ligands that induce the switch to anti-parallel topology although these ligands are less in relation to the ligands that induce the parallel topology. An example is the compound **IQc3d**, an indolo[3,2-*c*]quinoline derivative (Figure 22). Results from CD spectroscopy, when a preformed 21-nt hTelo DNA G4 was titrated with **IQc3d** in K^+^ solution, showed that this G4 ligand induces a conformational change from hybrid to an anti-parallel topology [298]. However, under the same conditions, **IQc3d** does not induce any change on the parallel G4 formed by the KRAS21R DNA sequence [297]. Another example of ligands able to switch hTelo DNA G4 to an antiparallel topology in K^+^ solution are bis(quinolinium) pyridine dicarboxamide (**PDC**) derivatives containing various coumarin or pyrene fluorophores (Figure 22) [340]. These ligands can induce conformational changes of hTelo hybrid DNA G4 structures (22AG, 25TAG, 24TTG) to an antiparallel topology, but had no effect on the other two topologies of hTelo G4, or on other types of G4-forming DNA sequences. Furthermore, two of the six compounds, **PDC-Ln-C2** (*n* = 1 or 2) demonstrated significant fluorescence enhancement (up to 180-fold) upon binding to several antiparallel and hybrid DNA G4s. In addition, the PDC-coumarin conjugate, **PDC-Ln-C2**, does not show significant fluorescence alone or in the presence of parallel G4, and therefore it is considered a selective light-up ligand for the antiparallel topology of telomeric G4.

Marchand and co-workers suggested that **PDC-360A** (Figure 17), converts h-Telo from a hybrid-type to an antiparallel topology in K^+^ solution [253]. However, it has been recently demonstrated that the ligand **PDC-360A** is also able to induce conformational switch from a hybrid to a parallel topology [183]. It should be noted that in this most recent study, the potassium concentration was lower, and the oligonucleotide sequence used was shorter (21 nt) than that used in the initial study (24 nt), which may have influenced the results. Moreover, the regioisomer **PDCd** (Figure 17) induced an interconversion of this G4 from hybrid to an antiparallel topology [183]. In addition, ligands **Phen-DC3** and **pyridostatin** (Figure 6) induce conformational changes of hTelo G4 to an antiparallel topology and the exclusion of a K^+^ cation upon binding. [253].

### 3.3. Alkylating G4 Ligands

**Figure 23:**
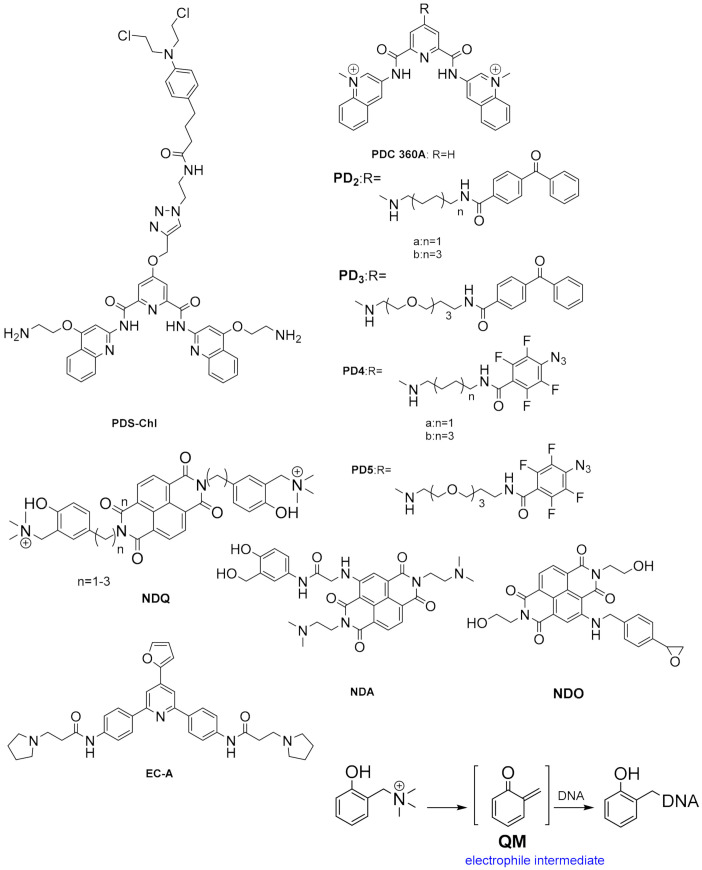
Alkylating G4 ligands and general mechanism of DNA alkylation by in situ generation of quinone methides.

The examples of ligands presented thus far focused on small molecules that bind to G4 in a reversible process through non-covalent interactions. However, in order to increase the selectivity and binding affinity of small molecules to the G4s, selective alkylation is another strategy that has been explored, especially in the last decade. Alkylating agents are the oldest class of anticancer drugs and the introduction of nitrogen mustards in the 1940s can be considered the origin of antineoplastic chemotherapeutics targeting nucleic acids. *N*-mustards are bifunctional alkylating agents, i.e., they have two electrophilic sites [341]. The potent G4 ligand pyridostatin was coupled with nitrogen mustard chlorambucil (**PDS-Chl**; Figure 23). Using different oligonucleotides, namely the G4-forming sequences (c-MYC and h-Telo), a double-stranded DNA (ds-DNA) and a mutated c-MYC sequence (c-MYC-MUT), which is unable to fold into a G4 conformation, the authors demonstrated that **PDS-Chl** alkylates only G4 structures at low μM concentrations. Incubation of c-MYC DNA G4 with **PDS-Chl**, at 37 °C, confirmed a dose-dependent alkylation of both adenines and guanines [342].

The MS fragmentation profile showed that the detected alkylated nucleobases were mono-adenine adducts (at N1 and exocyclic NH_2_), mono-guanine adducts (at N7 and N1), and bis-adducts with both adenine and guanine [342]. In addition, the bisquinolinum pyridine dicarboxamide G4 ligand **PDC 360A**, was tethered with two groups currently used for alkylating biomolecules, that is, benzophenone (**PD2** and **PD3**) and 4-azido-2,3,5,6-tetrafluoro-benzoic acid (**PD4** and **PD5**) with different linkers (Figure 23) [343]. Both groups can be excited by UVA irradiation (330–365 nm) to generate highly reactive intermediates evolving through diverse photochemical pathways. These derivatives were evaluated for their ability to form photoadducts with two well-known G4s, namely, the hTelo sequence 22AG and the *c-MYC* promoter sequence myc 22/G4T-G23T.

The highest yields were observed for the **PD2**, **PD4**, **PD3** and **PD5** derivatives (26–36% in K^+^, 13–29% in Na^+^). Moreover, it was found that DNA G4 alkylation by these compounds vary not only with the ionic conditions, but it is also strongly dependent on the nature of the spacer. The authors attribute the low efficiency of the alkyl chain C8 to its lipophilic character, which is therefore, less likely to be near the highly hydrated DNA structure. The benzophenone derivatives react exclusively with the loop thymine residues of 22AG: the ligand **PD2** alkylates the first and second loops (starting from the 5′ end), whereas compound **PD3** alkylates the three loops. In contrast, **PD4** and **PD5** adducts revealed exclusive alkylation of guanine residues G10 and G14, at the 5′ external G-quartet, regardless of the buffer.

G4 ligands, such as the naphthalene diimide core (NDI), coupled with a thermally activable alkylating moiety (e.g., a quinone methide), **NDQ**, an alcohol group, **NDA**, or with a classical alkylating agent (e.g., an oxirane), **NDO**, have also been reported (Figure 23) [344,345,346]. Quinone methides (**QM**) are highly electrophilic and have been extensively used as triggerable electrophiles to induce DNA alkylation (Figure 23). The potential of quinone methides in chemical biology has been recently reviewed elsewhere [347]. Upon light irradiation or thermal conditions *o*-quinone methide can be formed from (2-hydroxybenzyl)trimethylammonium (Figure 23) [344,345,347]. **NDQ**s’ revealed good reactivity on single nucleosides and selectivity towards hTelo G4, despite the low alkylation efficiency (15% yield) [345]. Furthermore, the Freccero group reported that compound **NDA** exhibited, under the same thermal conditions, a selective reactivity with G4 structure and with similar yields (16.8%). However, the formation of these resulting DNA adducts appeared to be reversible, which caused low cross-linking efficiency. In order to synthesize more stable and irreversible adducts, the NDI-oxirane derivative was developed (**NDO**) (Figure 23). Thanks to their peculiar characteristics, i.e., the electrophilic character of oxiranes, the resulting adducts revealed much higher thermal stability, an irreversible alkylation process, even under basic conditions, up to 90 °C. Results from mass-spectrometry showed a highly selective alkylation of adenine vs. guanine of the oligonucleotide hTelo{5′-d(AG3[T2AG3]3)-3′)} [345].

Very recently, to avoid off-target reactions that compromise the G4 selectivity, an alternative methodology for G4 alkylation using a furan-based light–triggered was proposed [348]. They synthesized the compound **EC-A** (Figure 23), and the activation was triggered by singlet oxygen generated in situ by light irradiation of a photosensitizer, the methylene blue. With this process, the pro-reactive furan can be oxidized into a reactive keto-enal, which rapidly reacts with exocyclic amines of DNA nucleobases. Therefore, the alkylation of G4 DNA relative to dsDNA was selectively achieved and furthermore, with a high yield. Mass analysis revealed the presence of mono and di-alkylation products.

## 4. Challenges in the Design of Therapeutically Useful G4 Targeting Small Molecules

Nowadays, there are no doubts on the capacity of G/C rich nucleic acids to form quadruplex structures in vivo, neither on the important biological roles they play in genome regulation across all organisms. Cumulative evidence supports the involvement of quadruplex nucleic acid structures in the regulation of gene replication, transcription, translation and epigenetic events, where G4s and iMs work together and function, in many cases, as protein recognition sites. Therefore, both G4s and iMs have been explored as drug targets, particularly for the treatment of cancer, but also of parasitic, bacterial or viral infections. Moreover, quadruplexes have also been recently proposed as drug targets in neurological disorders.

In the past decades, hundreds of quadruplex-interactive small molecules have been developed and shown, through in-vitro and in-vivo assays, to have potential to be developed as therapeutics (Table 1, Table 2 and Table 3), with three of these molecules reaching clinical trials for cancer therapy. One of the major challenges during the development of a drug is the achievement of target selectivity, and in the development of quadruplex targeting drugs that is not different. As shown throughout this overview, most quadruplex binders, although selective for quadruplexes in respect to other nucleic acid secondary structures, are unable to discriminate between quadruplex-forming sequences. A few recent studies indicate that the desired selectivity/specificity may be achievable by sacrificing binding affinity [349]. Moreover, recent advances and future studies on the understanding of the interplay between quadruplexes and proteins will open new perspectives on the structure of the target, shifting the focus from the individual quadruplex structure to the protein-quadruplex interaction region. However, one must not forget that in certain diseases in which multiple metabolic pathways and genes are unregulated, such as in cancer, this quadruplex targeting promiscuity may be an advantage.

The understanding of how these small molecules interact with individual quadruplexes and the effects they induce on the quadruplex structure upon ligand binding is of utmost importance for the development of more specific quadruplex targeting drugs or probes. Moreover, even considering the differences between the in-vitro conditions of these studies and the complex biological context in which quadruplexes are formed, the study of such molecules in the interaction with quadruplexes, is essential to unveil their biological functions. Currently, many ligand-G4 complexes have been resolved and their structures deposited in the Protein Data Bank. As shown in Figure 8, Figure 9 and Figure 10, most of these complexes are formed by compounds able to adopt a planar geometry to bind through π–π stacking to the external G-quartets and stabilize the G4.

However, not all quadruplex-interactive small molecules induce a stabilizing effect. As summarized in Table 6, most G4-stabilizers are iM-destabilizers, whereas other ligands induce different effects depending not only on the quadruplex structure, but also on the ligand concentration and conditions of the assay. Interestingly, the most recent studies are unravelling new chemotypes with preference to interact with iMs and control their stability. Moreover, many of these small molecules have also shown the capacity to induce G4 conformational switches in solution (Table 7 and Figure 21 and Figure 22), which could be exploited as nanosensors, probes or drugs interfering with G4 recognition by proteins. In this respect, whereas the present knowledge can be easily translated into novel nanotechnologies to be used in-vitro, its translation into new therapeutic agents or probes to recognize and study specific quadruplexes and their protein interactions requires additional studies. It is now necessary to understand if these molecules retain their capacity to induce the same effect in a cellular environment.

## 5. Conclusions

Overall, this updated overview on the therapeutic potential of quadruplex-interactive small molecules, their mode of binding and effects upon interaction with quadruplexes, contribute to put in perspective the challenges ahead on the design of therapeutic small molecules targeting G4 and/or iM nucleic acids. Major challenges include the understanding of the chemical features necessary to target a specific G4 or iM, and induce, in the biological environment, a specific effect on its topology. To achieve target specificity, the design of G4/iM ligands paradigm must evolve from the simple quadruplex binder and stabilizer. One promising and emerging area of research is the quadruplex-protein interactome. Adding another “layer” to the target may be the way to achieve the desired drug selectivity.

## Figures and Tables

**Table 7:** Other G4 ligands inducing topological switches.

Ligand (Chemotype)	Structure	Topology Switch			Refs.
**3,3-L2H2-6OTD** (Oxazole-based)	Figure 15	ssDNA	➔	antiparallel (ion free)	[257]
**RHPS4** (acridinium)	Figure 4	hybrid	➔	antiparallel (in K^+^)	[282]
**Berberine**	Figure 13	hybrid	➔	antiparallel (in K^+^)	[282]
**BRACO-19** (acridine)	Figure 4	hybrid	➔	antiparallel (in K^+^)	[282]
**pyridostatin**	Figure 6	hybrid	➔	antiparallel (in K^+^)	[253]
**PhenDC3** (phenanthroline)	Figure 6	hybrid	➔	antiparallel (in K^+^)	[253,282]
**PDC-360A** (pyridine-dicarboxamide)	Figure 6	hybrid	➔	antiparallel (in 10 mM K^+^)	[253]
		hybrid	➔	parallel (in 60 mM K^+^)	[183]
**PDCd** (pyridine-dicarboxamide)	Figure 17	hybrid	➔	antiparallel (in K^+^)	[183]

## Data Availability

Not applicable.
